# Targeting cancer stem cell pathways for cancer therapy

**DOI:** 10.1038/s41392-020-0110-5

**Published:** 2020-02-07

**Authors:** Liqun Yang, Pengfei Shi, Gaichao Zhao, Jie Xu, Wen Peng, Jiayi Zhang, Guanghui Zhang, Xiaowen Wang, Zhen Dong, Fei Chen, Hongjuan Cui

**Affiliations:** 1 State Key Laboratory of Silkworm Genome Biology, Southwest University, 400716 Chongqing, China; 2 Cancer Center, Medical Research Institute, Southwest University, 400716 Chongqing, China; 3 Department of Pharmaceutical Sciences, Eugene Applebaum College of Pharmacy and Health Sciences, Wayne State University, Detroit, MI 48201 USA

**Keywords:** Cancer stem cells, Cancer stem cells

## Abstract

Since cancer stem cells (CSCs) were first identified in leukemia in 1994, they have been considered promising therapeutic targets for cancer therapy. These cells have self-renewal capacity and differentiation potential and contribute to multiple tumor malignancies, such as recurrence, metastasis, heterogeneity, multidrug resistance, and radiation resistance. The biological activities of CSCs are regulated by several pluripotent transcription factors, such as OCT4, Sox2, Nanog, KLF4, and MYC. In addition, many intracellular signaling pathways, such as Wnt, NF-κB (nuclear factor-κB), Notch, Hedgehog, JAK-STAT (Janus kinase/signal transducers and activators of transcription), PI3K/AKT/mTOR (phosphoinositide 3-kinase/AKT/mammalian target of rapamycin), TGF (transforming growth factor)/SMAD, and PPAR (peroxisome proliferator-activated receptor), as well as extracellular factors, such as vascular niches, hypoxia, tumor-associated macrophages, cancer-associated fibroblasts, cancer-associated mesenchymal stem cells, extracellular matrix, and exosomes, have been shown to be very important regulators of CSCs. Molecules, vaccines, antibodies, and CAR-T (chimeric antigen receptor T cell) cells have been developed to specifically target CSCs, and some of these factors are already undergoing clinical trials. This review summarizes the characterization and identification of CSCs, depicts major factors and pathways that regulate CSC development, and discusses potential targeted therapy for CSCs.

## Introduction

Cancers are chronologic diseases that seriously threaten human life. Many strategies have been developed for cancer treatment, including surgery, radiotherapy, chemotherapy, and targeted therapy. Because of all these treatments, the incidence rate of cancer has been stable in women and has declined slightly in men in the past decade (2006–2015), and the cancer death rate (2007–2016) also declined.^1^ However, traditional cancer treatment methods are effective only for some malignant tumors.^2^ The main reasons for the failure of cancer treatment are metastasis, recurrence, heterogeneity, resistance to chemotherapy and radiotherapy, and avoidance of immunological surveillance.^3^ All these failures could be explained by the characteristics of cancer stem cells (CSCs).^4^ CSCs can cause cancer relapse, metastasis, multidrug resistance, and radiation resistance through their ability to arrest in the G0 phase, giving rise to new tumors.^5^ Therefore, CSCs could be considered the most promising targets for cancer treatment.

CSCs were first identified in leukemia and then isolated via CD34^+^ and CD38^−^ surface marker expression in the 1990s.^6,7^ CSCs expressing different surface markers, such as CD133, nestin, and CD44, have been subsequently found in many nonsolid and solid tumors, and these cells also form the bulk of the tumor.^8,9^ CSCs can generate tumors via the self-renewal and differentiation into multiple cellular subtypes.^10^ The activities of CSCs are controlled by many intracellular and extracellular factors, and these factors can be used as drug targets for cancer treatment.^11^ To understand the nature of CSCs, we summarized their characteristics, methods for identification and isolation, regulation and current research on targeting CSCs for cancer therapy both in basic research and clinical studies.

## The concept of CSCs

### Biological characteristics of CSCs

With the deepening of tumor biology research, clinical diagnosis and cancer treatment have significantly improved in recent years. However, the high recurrence rate and high mortality rate are still unresolved and are closely related to the biological characteristics of CSCs. With further understanding of CSC characteristics, research on tumor biology has entered a new era. Therefore, understanding the biological properties of CSCs is of great significance in the diagnosis and treatment of tumors.

CSCs have a strong self-renewal ability, which is the direct cause of tumorigenesis.^12^ CSCs can symmetrically divide into two CSCs or into one CSC and one daughter cell.^13^ CSCs expand in a symmetrical splitting manner to excessively increase cell growth, ultimately leading to tumor formation.^14^ CSCs isolated from original tumor tissue that were transplanted into severe combined immunodeficiency disease (SCID) mice then formed new tumors.^15^ CSCs and normal stem cells also share some of the same regulatory signaling pathways, such as the Wnt/β-catenin,^16^ Sonic Hedgehog (Hh),^17^ and Notch pathways, which are involved in the self-renewal process.^18^ In addition, other signaling molecules, such as PTEN and the polycomb family, also play important roles in the regulation of CSC growth.^19^ The regulation of CSC self-renewal is the key link to understanding tumorigenesis. These studies will provide a clear target for cancer treatment.

In addition to their self-renewal ability, CSCs also have the ability to differentiate into different cell types. Bonnet and Dick^7^ demonstrated in 1997 that CD34^+^/CD38^−^ leukemia stem cells (LSCs) have the ability to differentiate and proliferate in SCID mice. Brain CSCs isolated from patients are positive for the markers CD133 and nestin, which are the same markers as those of normal neuronal stem cells, but some cells lack surface markers for differentiation.^20^ Generally, various signaling pathways regulate the self-renewal and differentiation of normal stem cells to promote their proliferation and differentiation in a relatively balanced manner. Once the regulatory balance is destroyed, uncontrolled CSCs ultimately lead to tumorigenesis.^21^ CSCs also transdifferentiate into other multilineage cells to regulate tumorigenesis.^22^ Bussolati et al.^23^ found that renal CSCs differentiated into vascular endothelial cells (ECs) in the bulk of tumors formed in SCID mice after injection of human renal CSCs. Additionally, CSCs that differentiate into vascular ECs and promote angiogenesis have been found in a variety of cancers, such as glioblastoma^24^ and liver cancer.^25^

Metastasis refers to the process by which cancer cells travel from the primary site through lymphatic vessels, blood vessels, or the body cavity.^26^ Since stromal cells (such as granulocytes and macrophages) secrete signaling molecules in the tumor microenvironment (TME), these cells stimulate epithelial–mesenchymal transformation (EMT) to promote the invasion of tumor cells,^27^ which induce differentiated human mammary epithelial cells to form mammary glands.^28^ Activation of the RAS/MAPK (mitogen-activated protein kinase) signaling pathway transforms nontumorigenic CD44^−^/CD24^+^ breast cancer cells into tumorigenic CD44^+^/CD24^−^ breast cancer cells.^29^ A study showed that CSCs are closely related to EMT, and EMT is likely to be the basis for tumor invasion and metastasis. In addition, CD133^+^/CXCR4^+^ pancreatic cancer cells^30^ and CD44^+^/α2β^hi^1/CD133^+^ prostate cancer cells^31^ are also tumorigenic. Therefore, these studies indicate that CSCs play a crucial role in tumor metastasis and development.

Furthermore, understanding the mechanism of CSC drug resistance is vital for cancer treatment and preventing recurrence.^32^ CSCs efficiently express ATP-binding cassette (ABC) transporters (including MDR1 (ABCB1), MRP1 (ABCC1), and (ABCG2)), which are multidrug resistance proteins, and these proteins protect leukemia and some solid tumor cells from drug damage and induce drug resistance.^33^ According to previous studies, aldehyde dehydrogenase (ALDH), a marker in many CSCs,^34^ eliminates oxidative stress and enhances resistance to chemotherapeutic drugs, such as oxazolidine, taxanes, and platinum drugs.^35^ ALDH also removes free radicals induced by radiation and stimulates resistance to radiation.^35^ Inducing DNA damage and apoptosis through chemotherapy and radiotherapy are commonly used cancer treatments. However, CSCs can effectively protect cancer cells from apoptosis by activating DNA repair abilities.^36^

It is currently believed that CSCs are the key "seeds" for tumor initiation and development, metastasis, and recurrence.^37^ CSCs have evolved and are highly heterogeneous.^38^ Breast CSCs have different expression patterns of surface biomarkers, such as CD44^+^, CD24^−^, SP, and ALDH^+.^^29,34,39^ CD271^−^ or CD271^+^ melanoma stem cells can form tumors in SCID mice.^40^ The heterogeneity of CSCs has also been found in other cancers, including glioblastoma,^41^ prostate cancer,^42^ and lung cancer.^43^ The heterogeneity of CSCs is so complex that more effective biomarkers are needed to identify CSCs or distinguish the heterogeneity of CSCs.

### Isolation and identification of CSCs

It is known that the proportion of CSCs in tumor tissues is very low and generally accounts for only 0.01–2% of the total tumor mass. In addition, CSCs and normal stem cells also share similar transcription factors and signaling pathways. Therefore, it is more challenging to isolate and identify CSCs. However, an increasing number of techniques and means have emerged.

CSCs have been identified through different biomarkers in human cancers (Table 1). CSCs can be separated by combining specific biomarkers that are mostly located on the cell surface.^3^ The primary separation techniques are fluorescence-activated cell sorting (FACS) and magnetic-activated cell sorting (MACS).^44,45^ Since Dick JE first screened CSCs from leukemia by using FACS technology,^7^ FACS has become the most widely used technique for cell separation. It can perform multibiomarker sorting at one time and has high purity and strong specificity. MACS is a MACS technique. MACS separation is relatively simple, but the technique is cumbersome. Therefore, this method requires high activity of CSCs.^44,46^ These two methods are effective in separating CSCs from large numbers of cells.

**Table 1:** Various biomarkers of cancer stem cells in human cancers

Cancers	Markers	Function
Breast	CD29^+^^658^, CD49f^+^^659^, CD90^+^^660^, CD133^+^^661^, ALDH^+^^662^, ESA^+^/CD44^+^/CD24,^663^ CD44^+^/CD24^−^^664^	ALDH: An enzyme that plays a role in cell resistance^665^ CD44: A glycoprotein involves in cell migration and self-renewal^666^ CD90: A glycoprotein participates in T cell adhesion and signal transduction^667^ CD133: A transmembrane glycoprotein that maintains lipid composition in cell membranes^668^ CD24: A marker that promotes blood flow in the tumor during metastasis^669^ CD49f: A membrane proteins of the integrin family that plays an important role in cell surface adhesion and signaling^670^
Prostate	EpCAM^+^^671^, CD117^+^^672^, α2β1^+^^31^, ALDH^+^^42^, CD44^+^^673^, EZH2^+^^674^, CXCR4^+^^675^, E-cadherin^+^^676^, CD133^+^^677^	α2β1: A receptor involves in cell adhesion and recognition^31^ E-cadherin: It plays an important role in tumor migration and invasion^676^ CXCR4: CXC chemokine receptor works with CD4 protein to support HIV entry into cells^675^ EZH2: A member of the Polycomb family plays an vital role in the central nervous system^674^
Brain	CD49f^+^^678^, CD90^+^^679^, CD44^+^^680^, CD36^+^^681^, EGFR^+^^682^, A2B5^+^^683^, L1CAM^+^^684^, CD133^+^^41,685^	CD36: The main glycoprotein on the surface of platelet has an important function as an adhesion molecule^686^ EGFR: It binds to epidermal growth factor and promote proliferative migration in tumors^682^ A2B5: A ganglioside marker that identifies subpopulations of nerve cells in the central nervous system^687^ L1CAM: A adhesion molecule that plays an important role in the development of the nervous system include neuronal migration and differentiation^684^
Stomach	ALDH^+^^688^, CD44^+^^689^, CD44V8–10^+^^690^, CD133^+^^691^, CD24^+^^692^, CD54^+^^693^, CD90^+^^694^, CD49f^+^^678^ CD71^+^^695^, EpCAM^+^^696^	CD44V8–10: A variant of CD44 with a specific class of CSCs^690^ CD54: A class of adhesion molecules express in malignant tumor cells^693^
Colorectal	CD200^+^^697^, EpCAM^+^^698^, CD133^+^^699^, CD166^+^, CD206^+^^700^, CD44^+^^701^, CD49f^+^^678^, ALDH^+^^702^	CD200: A glycoprotein plays an important role in the regulation of immunosuppression and anti-tumor activity^703^ CD166: It binds to the T cell differentiation antigen CD6 and involves in cell adhesion and migration processes^704^ CD206: A mannose receptor involves in endocytosis, phagocytosis, and immune homeostasis^700^ EpCAM: It expresses on most normal epithelial cells and gastrointestinal cancers, and acts as a homotypic calcium-independent cell adhesion molecule^705^
Liver	CD24^+^^706^, CD133^+^^707^, CD13^+^^708^, CD44^+^^709^, CD206^+^^700^, OV-6^+^^708^, CD90^+^^710^, EpCAM^+^^711^	CD13: A receptor for human coronavirus strains, which is the main cause of upper respiratory tract infection and leukemia^712^ OV-6: A marker for rat oval cells and hepatic stem cells^708^
AML	CD34^+^, CD38^−^, CD90^+^, CD71^+^, CD19^+^, CD20^+^, CD44^+^, CD10^+^, CD45RA^+^, CD123^+^^15^	CD34: It plays a role in the attachment of stem cells to bone marrow extracellular or stromal cells^713^ CD38: An intracellular Ca^2+^ mobilization messenger, prognostic markers for patients with chronic lymphocytic leukemia^714^ CD71: A transferrin receptor is important for nerve development^715^ CD19: A class of signal transduction molecules regulate B lymphocyte differentiation^716^ CD20: The protein plays a role in the development and differentiation of B cells into plasma cells^717^ CD10: It inhibits a variety of peptide hormones, include glucagon, encephalin, oxytocin, and bradykinin^718^ CD45RA: A class of leukocyte activation regulators^719^ CD123: An interleukin-specific subunit of a heterodimeric cytokine receptor^720^
Melanoma	CD20^+^^721^, CD271^+,^^722^, ALDH^+^^723^, CD133^+^^724^	CD271: A nerve growth factor receptor mediates cell survival and cell death in nerve cells^725^
Bladder	CD44v6^+^^726^, CD44^+^^727^, ALDH^+^^728^	CD44v6: It involves in cell migration, cell adhesion^729^
Ovarian	CD24^+^^730^, ALDH^+,^^731^, CD44^+^/CD117^+^^732^, EpCAM^+^^733^, CD133^+^^734^	CD117: A class of transmembrane receptors is also known as stem cell factors^735^
Pancreas	ALDH^+^^736^, CD133^+^^30^, CD44^+^/CD24^+^/EpCAM^+^^17^, ABCG2^+^^737^, CXCR4^+,^^738^	ABCG2: A class of membrane proteins belongs to the ABC transporter superfamily that plays a role in the drug resistance properties of CSCs
HNSCC	ALDH^+^^739^, CD44^+,^^740^, CD166^+^^741^	
Gallbladder	CD44^+^/CD133^+^^742^	
RCC	CD133^+^^743^, ALDH^+,^^743^, CXCR4^+^^743^, CD44^+,^^744^, CD105^+^^23^	CD105: TGF receptor that involves in TGF-β signaling plays a role in angiogenesis^745^
Lung	CD166^+^^746^, CD90^+,^^747^, CD87^+^^748^, ALDH^+,^^749^, CD44^+^^750^, CD133^+^^751^	CD87: A receptor for urokinase plasminogen activator that affects many normal and pathological processes associates with cell surface plasminogen activation and local degradation of extracellular matrices^748^
Malignant mesothelioma	CD9^+^, CD24^+^, CD26^+^^752^	CD9: A glycoprotein plays a role in many cellular processes, includes differentiation, adhesion and signal transduction, and plays a key role in cancer cell movement and metastasis^753^ CD26: A class of serine exopeptidases is also an intrinsic membrane glycoprotein^754^
OSCC	CD44^+^/CD24,^−755^ ITGA7^+^^756^	ITGA7: A integrin plays a role in cell migration, morphogenesis, differentiation, and metastasis and participates in the process of differentiation and migration during myogenesis^757^
cSCC	CD44^+^^758^, CD133^+^^759^	
Esophageal	ITGA7^+^, CD44^+^, ALDH^+^, CD133^+^, CD90^+^^297^	
MM	CD138−, CD19^+^, CD27^+^^760,761^	CD138: A member of the Syndecan proteoglycan family that involves in cell proliferation, cell migration, and cell–matrix interactions^762^ CD27: A transmembrane glycoprotein involves in the regulation of B cell activation and immunoglobulin synthesis^763^
Cervix	ABCG2^+^, CD133^+^, CD49f^+764^, ALDH^+^^765^	
Nasopharyngeal	CD44^+^^766^, CD133^+^^767^, ALDH^+^^768^, CD24^+^^769^	
Laryngeal	ALDH^+^, CD44^+^^770^, CD133^+^^771^	

*AML* acute myeloid leukemia, *HNSCC* head and neck squamous cell carcinoma, *RCC* renal cell carcinoma, *OSCC* oral squamous cell carcinoma, *cSCC* cutaneous squamous cell carcinoma, *MM* multiple myeloma, *ALDH* aldehyde dehydrogenase, *EpCAM* epithelial cellular adhesion molecule

Additionally, there are other ways to separate CSCs from tumors. In 1996, Dr. Goodell observed that after adding Hoechst 33342 to a culture of bone marrow cells, a few cells did not accumulate dyes, and he claimed that these few cells were side population (SP) cells. Therefore, SP cells can be separated by fluorescence screening after the outflow of Hoechst 33342. Recently, SP cells have been identified in various normal tissues and tumor cells. SP cells have high homology, self-renewal and multidirectional differentiation potential.^47,48^ Some reports have shown that ABCG2 is highly expressed in SP cells.^47,49^ ABCG2 is highly related to the drug resistance of CSCs and is used as a phenotypic marker for CSCs,^50,51^ including ovarian cancer,^52^ AML,^53^ breast cancer,^54^ lung cancer,^55^ nasopharyngeal carcinoma,^56^ and hepatocellular carcinoma (HCC).^57^ Montanaro et al.^58^ explored the optimal concentration of Hoechst 33342 to reduce the toxic effect. The SP sorting method has universal applicability in the separation and identification of CSCs, especially CSCs with unknown cell surface markers, and is an effective method for CSC research.

The colony-forming ability of CSCs is also used for separation and identification.^59^ After digestion of the tumor tissues into single cells, low-density cell culture can be conducted in serum-free medium containing epithelial growth factor (EGF) and basic fibroblast growth factor (FGF).^60^ Under this condition, a single CSC will form a cell colony or sphere. Taylor et al.^61^ successfully isolated CSCs from a variety of neurological tumors by using this colony formation assay. However, the cell purification rate is low, and the CSC specificity is poor in this assay. The in vivo limited dilution assay (LDA) can be used for assessing CSC activity. After low-density transplantation of immune-deficient mice with the limiting dilution method, CSCs can be identified by ELDA software analysis, and this method is affected by cell density and the microenvironment in mice.^62^

Traditional chemotherapeutic drugs mainly affect cancer cells, but CSCs are mostly arrested in the G0 phase and are relatively static, thus evading the killing effect of chemotherapeutic drugs.^63^ Hence, the drug-resistant characteristics of CSCs can be used to isolate and identify CSCs.^64^ Previous studies have shown that radiotherapy combined with hypoxic culture can also be used to enrich CSCs.^65^ In addition, the separation of CSCs can also be accomplished by physical methods. Hepatoma stem cells can be isolated from rat liver cancer tissue by Percoll density gradient centrifugation; a cell fraction with a high nuclear-to-cytoplasmic ratio is obtained.^66^ Recently, Rahimi et al.^67^ used the miR-302 host gene promoter to overexpress neomycin in cancer cells and selected and collected neomycin-resistant CSCs.

## Factors regulating CSCs

CSCs can originate from at least four cell types, including normal stem cells, directed group progenitor cells, mature cells, and the fusion of stem cells and other mutant cells.^68^ Therefore, transformed CSCs from normal cells require multiple gene mutations, epigenetic changes, uncontrolled signaling pathways, and continuous regulation of the microenvironment. It is currently believed that there are many similarities between CSCs and embryonic stem (ES) cells, especially regarding their ability to grow indefinitely and self-renew, signaling pathways and some transcription factors. In addition, CSCs exist in the supporting microenvironment, which is vital for their survival. Moreover, the complex interaction between CSCs and their microenvironment can further regulate CSC growth. This section will discuss the effects of transcription factors, signaling pathways, and the microenvironment on CSC survival, apoptosis, and metastasis.

### Major transcription factors in CSCs

Generally, stem cells have at least two common characteristics: the ability to self-renew and the potential to differentiate into one or more specialized cell types.^69^ Somatic cells can be reprogrammed to become induced pluripotent stem cells by transient ectopic overexpression of the transcription factors Oct4, Sox2, Nanog, KLF4, and MYC.^70–72^ In addition, there are some similarities between CSCs and ES cells. It is reasonable that some embryonic transcription factors can be re-expressed or reactivated in CSCs.^69^ Therefore, these transcription factors play a very important role in the regulation of CSC growth.

Oct4, a homeodomain transcription factor of the Pit-Oct-Unc family, is recognized as one of the most important transcription factors.^73^ Recently, Oct4 has emerged as a master regulator that controls pluripotency, self-renewal, and maintenance of stem cells.^74^ Some studies have reported that Oct4 is highly expressed in CSCs.^70,73^ High expression of Oct4 is positively correlated with glioma grades^75^ and promotes self-renewal, chemoresistance, and tumorigenicity of HCC stem cells.^76^ High expression of Oct4 is also observed in breast CSC-like cells (CD44^+^/CD24^−^).^77^ Cisplatin, etoposide, adriamycin, and paclitaxel γ-irradiation upregulate the expression of Oct4 in lung cancer cells, and CD133^+^ cells are more resistant to drug treatments than CD133^−^ cells.^78^ Data also show that Oct4 expression is associated with poor clinical outcome in hormone receptor-positive breast cancer.^79^ Knockdown of Oct4 also reduces the stemness of germ cell tumors.^80^ Hence, these studies have proven that Oct4 is a pluripotent factor in CSCs.

Sox2 belongs to the family of high-mobility group transcription factors and plays a significant function in the early development and maintenance of undifferentiated ESCs. It is also one of the key transcription factors in CSCs. Rodriguez-Pinilla et al.^81^ found that increased expression of Sox2 in basal-like breast cancer may help to characterize poorly differentiated/stem cell phenotypes.^82^ Hagerstrand et al.^82^ also found that a high level of Sox2 can induce xenograft glioma. Further studies showed that knockout of Sox2 inhibits glioblastoma cell proliferation and tumorigenicity, which suggests that Sox2 is the basis for maintaining the self-renewal ability of tumor-initiating cells (TICs).^83^ Sox2 also maintains the self-renewal of TICs in osteosarcomas, and downregulation of Sox2 drastically decreases its transformative characteristics and tumorigenesis ability in vitro. Furthermore, osteosarcoma cells that lose Sox2 cannot form osteospheres and differentiate into mature osteoblasts any longer.^84^ Sox2 is found in invasive cutaneous squamous cell carcinoma (SCC) and promotes the metastasis of cancer cells.^85^ These studies suggest that Sox2 promotes self-renewal and tumorigenesis and inhibits differentiation in CSCs.

Nanog, a differentiated homeobox (HOX) domain protein that was first discovered in ESCs, has typical self-renewal and multipotent transcriptional regulatory functions.^86^ Although Nanog is silenced in normal somatic cells, abnormal expression has been reported in human cancers, such as breast cancer, cervical cancer, brain cancer, colon cancer, head and neck cancer, lung cancer, and gastric cancer.^86–90^ Compared to levels in benign tissues, Nanog messenger RNA (mRNA) is elevated in malignant tumors. In a number of patients with colorectal cancer (*n* = 175), high Nanog protein is associated with lymph node positivity and Dukes grade.^91^ Similarly, overexpression of Nanog in colorectal CSCs promotes colony formation and tumorigenicity in vivo.^92^ In addition, gastric cancer patients with high Nanog levels have a lower 5-year survival rate.^88^ The expression level of Nanog is increased in HCC cell lines and primary tumors and is associated with advanced diseases (tumor node metastasis (TNM) stage III/IV).^93^ Through the study of prostatic cell lines, xenografts and primary tumors, it was found that Nanog short hairpin RNA inhibits the formation of primary prostate cancer cells (PCA) spheres, clonal growth, and tumorigenesis.^94^ In 43 cases of pancreatic cancer tissue microarray analysis, Kaplan–Meier analysis showed that high expression of Nanog (and Oct4) predicted worse prognosis and was negatively correlated with patient survival.^95^ These studies indicate that Nanog plays an important role in regulating the self-renewal and proliferation of CSCs.

KLF4 is expressed in many tissues and plays an important role in many different physiological processes. As a bifunctional transcription factor, KLF4 activates or inhibits transcription according to different target genes and utilizing different mechanisms. KLF4 can play an oncogenic or anticancer role, depending on the type of cancer involved. For example, KLF4 is an anticancer factor in the intestinal epithelium and gastric epithelium.^96^ The expression of KLF4 is downregulated with hypermethylation and loss of heterozygosity in colorectal CSCs and gastric CSCs.^97^ Downregulation of KLF4 is also found in other cancers, such as non-small-cell lung carcinoma,^98^ liver cancer,^99^ leukemia,^100^ anaplastic meningioma,^101^ bladder cancer,^102^ and esophageal cancer.^103^ Although these data clearly demonstrate that KLF4 plays an anticancer role in those cancers, KLF4 may also be an oncogene, which was demonstrated for the first time in nearly a decade.^104^ Overexpression of KLF4 in transformed rat renal epithelial cells induces tumorigenesis of laryngeal SCC.^105^ In addition, depletion of KLF4 inhibits melanoma xenograft growth in vivo.^106^ High expression of KLF4, an oncogene in human breast CSCs, is correlated with an aggressive phenotype in canine mammary tumors.^107^ These studies suggest that KLF4 has different functions in different CSCs.

MYC has three family members (C-Myc, N-Myc, and L-Myc, which are encoded by the proto-oncogene family and are essential transcription factors in the DNA-binding proteins of the basic helix–loop–helix (bHLH) superfamily). MYC regulates a large number of protein-coding and noncoding genes and coordinates various biological processes in stem cells, such as cell metabolism, self-renewal, differentiation, and growth.^108,109^ Although the *MYC* gene is one of the most commonly activated oncogenes that is involved in the pathogenesis of human cancer, overexpression of MYC alone is surprisingly unable to induce the transformation of normal cells into tumor cells. The overexpression of MYC in normal human cells may be ineffective or highly destructive, resulting in stagnation of proliferation, aging, or apoptosis.^110^ MYC is usually deregulated in human cancers, plays an important role in maintaining the number of invasive CSCs,^111^ and is also one of the most effective oncogenes for detecting the cell transformation phenotype in vitro and in vivo. Previous studies have shown that deletion of the tumor suppressor gene *p53* and *MYC* synergizes to induce hepatocyte proliferation and tumorigenesis.^112^ In addition to p53 deletion, overexpression of Bcl-2 and Bmi-1 and loss of p19ARF also assist MYC in regulating the survival and proliferation of CSCs.^113^ The expression of the three members of the MYC family is different in different tumors, such as C-MYC in leukemia and tongue SCC stem cells^114,115^ and N-MYC in small-cell lung cancer, prostate cancer, neuroblastoma, and medulloblastoma.^116,117^ L-MYC is expressed in hematopoietic malignancies.^118^ In addition, inactivation of MYC results in HCC stem cells differentiating into hepatocytes and biliary duct cells to form bile duct structures, which might be associated with the loss of the tumor marker α-fetoprotein and increased expression of cytokeratin 8, hepatocyte markers, carcinoembryonic antigen, and the liver stem cell marker cytokeratin 19.^119^ Studies have also shown that MYC is highly expressed in glioblastoma multiforme stem cells and induces cell proliferation and invasion and inhibits apoptosis.^111^ Increased copy number of the *MYC* gene in human and mouse prostate CSCs has also been found.^120^ These studies indicate that MYC induces tumorigenesis with the help of other factors.

### Major signaling pathways in CSCs

Many signaling pathways that contribute to the survival, proliferation, self-renewal, and differentiation properties of normal stem cells are abnormally activated or repressed in tumorigenesis or CSCs. Many endogenous or exogenous genes and microRNAs regulate these complex pathways. These signaling pathways can also induce downstream gene expression, such as cytokines, growth factors, apoptosis genes, antiapoptotic genes, proliferation genes, and metastasis genes in CSCs. These signaling pathways are not a single regulator but interwoven networks of signaling mediators to regulate CSC growth. Therefore, this section will describe how signaling pathways regulate CSC growth.

#### Wnt signaling pathway in CSCs

**Fig. 1:**
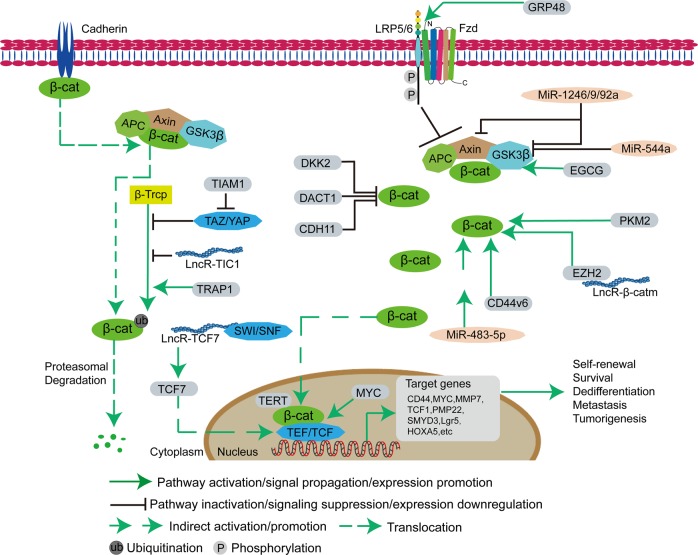
Wnt/β-catenin pathway in cancer stem cells. The canonical Wnt/β-catenin pathway regulates the pluripotency of CSCs and determines the differentiation fate of CSCs. In the absence of Wnt signaling, β-catenin is bound to the Axin complex, which contains APC and GSK3β, and is phosphorylated, leading to ubiquitination and proteasomal degradation through the β-Trcp pathway. However, the complex (TAZ/YAP), the long noncoding RNA TIC1 and proteins (TRAP1 and TIAM1) regulate the β-Trcp pathway. In the presence of Wnt signaling, the binding of LRP5/6 and Fzd inhibits the activity of the Axin complex and the phosphorylation of β-catenin, which makes β-catenin enter the nucleus, and then bind to TEF/TCF to form a complex, which then recruits cofactors to initiate downstream gene expression. Some proteins (DKK2 (Dickkopf-related protein 2), DACT1, CDH11, GECG, PKM2, EZH2, CD44v6, MYC, and TERT), microRNAs (miR-1246, miR-9, miR-92a, miR-544a, and miR-483-5p), and long noncoding RNAs (lncR-β-catm and lncR-TCF7) regulate the activation of the Wnt/β-catenin pathway in CSCs

Wnts include large protein ligands that affect diverse processes, such as the generation of cell polarity, and cells fate.^121^ The Wnt pathway is highly complex and evolutionarily conserved and includes 19 Wnt ligands and more than 15 receptors.^122^ The Wnt signaling pathway can be divided into canonical Wnt signaling (through the FZD-LRP5/6 receptor complex, leading to derepression of β-catenin) and noncanonical Wnt signaling (through FZD receptors and/or ROR1/ROR2/RYK coreceptors, activating PCP, RTK, or Ca^2+^ signaling cascades).^123^ In canonical Wnt signaling, in the absence of Wnt ligands (inactive Wnt signaling state, Fig. 1, left), β-catenin is phosphorylated by glycogen synthase kinase 3β (GSK3β), which leads to β-catenin degradation via β-TrCP200 ubiquitination and inhibits translocation of β-catenin from the cytoplasm to the nucleus.^124^ In contrast, in the presence of Wnt ligands (e.g., Wnt3a and Wnt1), the ligands combine with Fzd receptors and LRP coreceptors (active Wnt signaling, Fig. 1, right). LRP receptors are phosphorylated by GSK3β and CK1α.^125^ β-Catenin is released from the Axin complex to enter the nucleus. In addition, β-catenin combines with LEF/TCF and enhances the recruitment of histone-modifying coactivators, such as BCL9, Pygo, CBP/p300, and BRG1, to activate transcription. Noncanonical Wnt signaling does not involve β-catenin. During Wnt/PCP signaling, Dvl is activated through binding of Wnt ligands and the ROR-Frizzled receptor.^126^ Dvl inhibits the binding of the small GTPase Rho and the cytoplasmic protein DAAM1.^127^ The small GTPases Rac1 and Rho together trigger ROCK (Rho kinase) and JNK (c-Jun N-terminal kinase). This results in cytoskeletal rearrangement and/or transcriptional responses.^128^ Wnt/Ca^2+^ signaling is activated by G protein-triggered phospholipase C activity, which results in intracellular calcium flux and downstream calcium-dependent cytoskeletal and/or transcriptional responses.^129,130^

Aberrant Wnt signaling is found in many cancers, such as invasive ductal breast carcinomas,^131^ colorectal cancer,^132^ papillary thyroid cancer,^133^ esophageal cancer,^134^ and colorectal cancer.^135^ The activation of Wnt signaling is different in different tumors. Some Wnt activation is caused by mutations in Wnt components, such as Axin mutation in gastrointestinal cancers,^136^ APC mutation in colorectal cancer,^137^ and β-catenin mutation in gastric cancer and liver cancer.^138,139^ GSK3 genes are critical for β-catenin regulation; therefore, many researchers expect the occurrence of GSK3 mutations, but GSK3 mutations are not correlated with cancer occurrence. In addition, some genes (pyruvate kinase isozyme M2 (PKM2) in breast cancer^140^ and telomerase reverse transcriptase (TERT) in prostate cancer^141^) and microRNAs (miR-164a in colorectal cancer^142^ and miR-582-3p in non-small-cell lung cancer^143^) inhibit the activity of APC, Axin, and GSK3β to promote the accumulation of β-catenin in the cytoplasm.

Stem cell signaling pathways and transcriptional circuits are related to the alteration or reactivation of signaling pathways.^144^ Tumor dormancy is a lag phenomenon in tumor growth. Dormancy may occur during primary tumor formation or in the diffusion of some of the constituent tumor cells. However, primary tumor dormancy and metastatic dormancy seem to be different processes.^145^ In some cases, cells in the TME produce cytokines, such as Wnt proteins, secreted inhibitors of bone morphogenetic protein (BMP), and Delta, which activate the signaling pathway to maintain the self-renewal ability of CSCs.^146^ Activation of Wnt induce the transformation of dormant CSCs into active CSCs to promote cell cycle progression through β-catenin, increasing the expression of downstream cyclin D1 and MYC, and MYC also promotes the expression of the polycomb repressor complex 1 component Bmi-1 and induces the combination E2F with cyclin E.^147^ The extracellular matrix (ECM) protein tenascin C often exists in the gap of stem cells, which supports the cell cycle in breast cancer cells by increasing Wnt signals.^148^ In addition, aberrant Wnt signaling has also been observed in the self-renewal of CSCs (Fig. 1). Many reports have proven that numerous proto-oncogenes stimulate this process through the Wnt signaling pathway.^135^ PKM2 catalyzes the last step of glycolysis and plays an essential role in the proliferation of breast CSCs by associating with increased β-catenin levels at regions “−410 to 180 and −2250 to 2000”.^140,145,149^ Enhancer of zeste homolog 2 (EZH2), a key component of the polycomb PRC2 complex, promotes self-renewal of CSCs by activating β-catenin.^150^ Moreover, TERT, an RNA-dependent DNA polymerase, acts as a cofactor and forms a complex with β-catenin to activate Wnt downstream targets in prostate CSCs.^141^ Capillary morphogenesis gene 2 increases the expression of nuclear β-catenin to regulate the self-renewal and tumorigenicity of gastric CSCs,^151^ and SMYD3, which is located downstream of the Wnt pathway, has a similar effect.^152^ In addition, long noncoding RNAs and microRNAs also promote self-renewal of CSCs through the Wnt signaling pathway. LncTCF7 recruits the SWI/SNF complex to regulate the expression of the TCF7 promoter in liver CSCs.^153^ Lnc-β-Catm associates with the methyltransferase EZH2 to suppress the ubiquitination of β-catenin and promote its stability,^154^ and LncTIC1 interacts with β-catenin and maintains its stability, activating Wnt/β-catenin signaling.^155^ MicroRNA-1246, miR-19, and miR-92a suppress the expression of AXIN and GSK3β in CSCs.^156^ MicroRNA-544a downregulates GSK3β in lung CSCs.^157^ MicroRNA-483-5p upregulates the expression of β-catenin in gastric CSCs.^158^ In addition, there are still many genes, microRNAs, and noncoding RNAs in CSCs’ self-renewal through the Wnt signaling pathway.

Wnt signaling also plays an important role in the dedifferentiation of CSCs. HOXA5, which is a member of the HOX family, induces the differentiation of colorectal CSCs. However, Wnt indirectly suppresses indirectly via MYC, which is an important direct target of β-catenin/TCF in the intestine.^159^ PMP22, an integral membrane glycoprotein in myelin in the peripheral nervous system, induces the differentiation of gastric CSCs, but its mRNA level declines with activation of the Wnt/β-catenin pathway.^160^ Moreover, TRAP1, a component of the HSP90 (heat-shock protein 90) chaperone family, inhibits the differentiation of colorectal carcinoma stem cells by modulating β-catenin ubiquitination and phosphorylation.^161^ Lgr5, a member of the G protein-coupled receptor (GPCR) family of proteins, is located downstream of the Wnt signaling pathway and restrains the differentiation of esophageal SCC stem cells.^162^

Wnt signaling also plays an important role in regulating CSC apoptosis. Dickkopf-related protein 2 induces G0/G1 arrest and cell apoptosis by suppressing β-catenin activity in breast CSCs.^163^ DACT1, a homolog of Dapper that is located at chromosomal region 14q23.1, promotes apoptosis in breast CSCs by antagonizing the Wnt/β-catenin signaling pathway.^164^ Cadherin-11, a proapoptotic tumor suppressor, reduces the level of active phospho-β-catenin (ser552) to induce apoptosis in colorectal CSCs.^165^ Epigallocatechin-3-gallate increases apoptosis by degrading β-catenin in lung CSCs.^166^ The small-molecule inhibitor CWP232228 antagonizes the binding of β-catenin to TCF in the nucleus to induce apoptosis in liver CSCs.^167^ In addition, temozolomide combined with miR-125b significantly induces apoptosis by targeting the Wnt/β-catenin signaling pathway in glioma stem cells.^168^

Wnt/β-catenin signaling has been implicated in CSC-mediated metastasis.^169^ In the cytomembrane, Frizzled8 promotes bone metastasis in prostate CSCs.^170^ The leucine-rich repeat containing GPCR4 (LGR4, or GPR48), together with its family members LGR5/6, binds to R-spondins 1–4 and leads to Wnt3A potentiation, activating Wnt signaling in breast CSCs.^171,172^ Increased levels of CD44v6 mRNA in human pancreatic CSCs, lung CSCs, and colon CSCs promote migration and metastasis through the activation of β-catenin.^173–175^ In the cytoplasm, TAZ/YAP interacts directly with β-catenin and restricts β-catenin degradation,^176^ but TIAM1 antagonizes TAZ/YAP accumulation and translocation from the cytoplasm to the nucleus.^177^ Moreover, CDH11 inhibits the migration and invasion of colorectal CSCs by inhibiting Wnt/β-catenin and AKT/RhoA signaling.^165^ Wnt signaling decreases the expression of HOXA5 to promote CSC metastasis.^159^ These data suggest that amplified Wnt signaling is important for self-renewal, dedifferentiation, apoptosis inhibition, and metastasis of CSCs.

#### Notch signaling pathway in CSCs

The Notch signaling pathway consists of the Notch receptor, Notch ligand (DSL protein), CSL (CBF-1, suppressor of hairless, Lag), DNA-binding protein, other effectors, and Notch regulatory molecules. In 1917, studies discovered the Notch gene in a mutant Drosophila. Mammals have four Notch receptors (Notch1–4) and five Notch ligands (Delta-like 1, 3, and 4, Jagged 1, and Jagged 2).^178^ Notch and DSL ligands are transmembrane proteins that mediate communication between neighboring cells. Under physiological conditions, the ligand binds to a Notch receptor that is expressed on neighboring cells in a juxtacrine manner, thereby triggering proteolytic cleavage of the intracellular domain (ICD) of Notch and its translocation into the nucleus to bind to the transcription factor CSL, forming the NICD/CSL transcriptional activation complex, which activates target genes of the bHLH transcription inhibitor family, such as HES, HEY, and HERP.^179,180^

The Notch pathway regulates cancer cells in many tumors, such as glioblastoma, leukemia, and those of the breast, pancreas, colon, and lung, among others.^181^ Different tumors and tumor subtypes express different Notch ligands and receptors. Therefore, Notch is known to function as both an oncogene and a suppressive gene. As an oncogene, Notch is overexpressed in gastric cancer,^182^ breast cancer,^183^ colon cancer,^184^ and pancreatic cancer. In contrast, Notch expression is downregulated in prostate cancer,^185^ skin cancer,^186^ non-small-cell lung cancer,^187^ liver cancer,^188^ and some breast cancers.^189^ Whether Notch acts as an oncogene or a tumor suppressor gene is determined by the microenvironment.^190^ Moreover, post-translational modifications of Notch receptors change their affinity for ligands and their intracellular half-lives.^191^

Many studies on the Notch pathway in CSCs have shown that activation of Notch promotes cell survival, self-renewal, and metastasis and inhibits apoptosis. Aberrant Notch signaling (Notch1 and Notch4) promotes self-renewal and metastasis of breast and HCC stem cells.^192,193^ However, microRNA-34a downregulates Notch1.^194^ Similarly, abundant Delta-like ligand 4 (DLL4) also promotes tumor angiogenesis and metastasis in gastric CSCs.^195^ Delta-like 1 activation of Notch1 signaling requires the assistance of the actin-related protein 2/3 complex to maintain the stem cell phenotype of glioma-initiating cells.^196^ Additionally, some intracellular genes also regulate the Notch signaling pathway. For example, MAP17 (DD96, PDZKIP1), a nonglycosylated membrane-associated protein, is located on the plasma membrane and the Golgi apparatus. MAP17 interacts with NUMB through the PDZ-binding domain to activate the Notch pathway in cervical CSCs.^197^ Inducible nitric oxide synthase promotes the self-renewal capacity of CD24^+^CD133^+^ liver CSCs through TACE/ADAM17 activation to regulate Notch1 signaling.^198^ Moreover, tumor necrosis factor-α (TNFα) enhances the CSC-like phenotype by activating Notch1 signaling in oral SCC cells.^199^ Overexpression of PER3 decreases the expression of Notch1 and Jagged 1 in colorectal CSCs.^200^ In addition, KLF4 and BMP4 also increase Notch1 and Jagged 1 in breast CSCs to regulate cell migration and invasion.^201,202^ BRCA1 is a key regulator of breast cancer cell differentiation; however, it is localized to a conserved intronic enhancer region within the Notch ligand Jagged 1 gene to maintain the stemness of breast CSCs.^203^ Similarly, increased Gli3 also promotes cell proliferation and invasion in oral SCC by increasing Notch2.^204^ Hypoxia/hypoxia-inducible factor (HIF)-induced migration and invasion is a well-known phenomenon that has been reported in numerous CSCs.^205^ Notch1 can induce the migration and invasion of ovarian CSCs in the absence of hypoxia.^206^ Hypoxia-induced Jagged 2 activation enhances cell invasion of breast CSCs^207^ and lung CSCs.^208^ Moreover, HIF-1α/2α regulates self-renewal and maintenance of glioblastoma stem cells.^209^ In addition, increased miR-200b-3p decreases Notch signaling to promote pancreatic CSCs to become asymmetric.^210^ MiR-26a directly targets Jagged 1 to inhibit osteosarcoma CSC proliferation.^211^ These studies indicate that Notch plays an important role in regulating the self-renewal, growth, and metastasis of CSCs.

#### Hh signaling pathway in CSCs

The Hh signaling pathway consists of ligands and receptors. The Hh signaling network is very complex, including extracellular Hh ligands, the transmembrane protein receptor PTCH, the transmembrane protein SMO, intermediate transduction molecules, and the downstream molecule GLI.^212^ The components of the Hh signaling pathway play different roles. The membrane protein SMO plays a positive regulatory role, while the transmembrane protein PTCH plays a negative regulatory role. PTCH has two subtypes, PTCH1 and PTCH2,^213^ and there is 73% homology between the two subtypes. GLI, an effector protein, has three subtypes, Gli1, Gli2, and Gli3, in vertebrates,^214^ and these effector proteins have different functions. Gli1 strongly activates transcription, while Gli3 inhibits transcription.^215^ Gli2 has dual functions of activating and inhibiting transcription but mainly functions as a transcriptional activator.^216,217^ Numerous studies have confirmed that Hh signaling is involved in embryonic development and the formation of the nervous system, skeleton, limbs, lung, heart, and gut.^218^ As an extracellular signaling pathway, in the presence of ligand signals, Hh ligands bind to PTCH receptors on target cell membranes and initiate a series of intracellular signal transduction processes.^219^ When there is no ligand signal, the transmembrane receptor PTCH on the target cell membrane binds to SMO and inhibits SMO activity, which prevents signaling.^220^ When the Hh ligand is present, it binds to PTCH, which changes the spatial conformation of PTCH, removing the inhibition of SMO activating the transcription factor GLI and inducing it to enter the cell nucleus, where GLI regulates cell growth, proliferation, and differentiation.^221^

Studies have confirmed that abnormal activation of the Hh signaling pathway can be found in human cancers,^222^ such as breast cancer,^223^ lung cancer,^224^ bladder cancer,^225^ pancreatic cancer,^226^ chondrosarcoma,^227^ rhabdomyosarcoma,^228^ neuroblastoma,^229^ medulloblastoma,^230^ and gastric cancer.^231^ However, activation of Hh signaling is different in different tumors. Gorlin syndrome (basal cell nevus syndrome), an autosomal dominant condition, is associated with germline loss of the PTCH1 gene. This condition is very common in basal cell carcinoma, rhabdomyosarcoma, and medulloblastoma.^232,233^ Other Hh pathway components are also mutated in human cancers, such as Gli1 and Gli3 mutations in pancreatic adenocarcinoma, Gli1 gene amplification in glioblastoma, and SUFU (suppressor of fused) mutations in medulloblastoma.^234,235^ In addition, other genes also regulate the Hh signaling pathway. Speckle-type POZ protein, an E3 ubiquitin ligase adaptor, inhibits Hh signaling by accelerating Gli2 degradation in gastric cancer.^236^

**Fig. 2:**
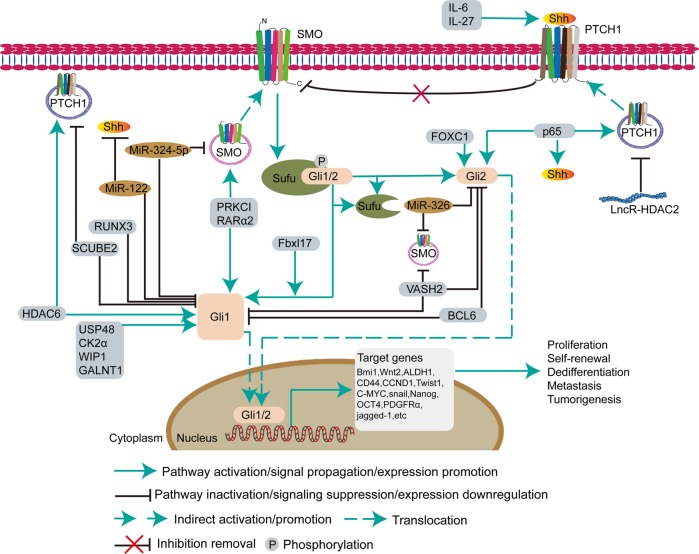
Hedgehog signaling pathway in cancer stem cells. The Hedgehog pathway plays a key role in stem maintenance, self-renewal, and regeneration of CSCs. The secreted Hh protein acts in a concentration- and time-dependent manner to initiate a series of cell responses, such as cell survival, proliferation, and differentiation. After receiving the Shh signal, the transmembrane protein receptor PTCH relieves the inhibition of the transmembrane protein SMO, which induces Gli1/2 to detach from SUFU and enter the nucleus to regulate downstream gene transcription. During activation of the Hh pathway, some proteins (IL-6, IL-27, Fbxl17 (F-box and leucine-rich repeat protein 17), PPKCI, RARα2, RUXN3, SCUBE2, HDAC6 (histone deacetylase 6), USP48, CK2α, WIP1, GALNT1, VASH2 (Vasohibin 2), BCL6, FOXC1 (forkhead box C1), and p65), microRNAs (miR-324-5p, miR-122, and miR-326), and the long noncoding RNA HDAC2 are involved in the Hedgehog pathway to affect CSC growth

Hh signaling plays distinct functions in different types of cancer.^237^ During tumor development, Hh signaling has three major roles: driving tumor development, promoting tumor growth, and regulating residual cancer cells after therapy. Based on these functions, the aberrant Hh pathway plays a causal role in CSCs^238,239^ (Fig. 2). The expression level of Hh signaling components is relatively high in CSCs. For example, Hh signaling promotes the maintenance, proliferation, self-renewal, and tumorigenicity of lung adenocarcinoma stem cells.^240^ In CD133^+^ glioma stem cells, SMO, GLI, and PTCH promote cell proliferation, self-renewal, migration, and invasion. The expression of Gli1, PTCH1, and PTCH2 is regulated by histone deacetylase 6.^241^ USP48 activates Gli-dependent transcription by stabilizing the Gli1 protein in glioma stem cells.^242^ The protein kinase CK2α enhances Gli1 expression and its transcriptional activity in lung CSCs.^243^ WIP1 (PPM1D), a nuclear Ser/Thr phosphatase, also enhances the function of Gli1 by increasing its transcriptional activity, protein stability, and nuclear localization in breast CSCs and medulloblastomas.^244,245^ F-box and leucine-rich repeat protein 17 mediates the release of Gli1 from SUFU for proper Hh signal transduction in medulloblastoma stem cells.^246^ Moreover, retinoic acid receptor α2 (RARα2) upregulates the expression of SMO and Gli1 in CD138^+^ multiple myeloma stem cells.^247^ PRKCI, which is regulated by miR-219 in tongue SCC,^248^ has a similar function as RARα2 in maintaining a stem-like phenotype in lung SCC cells.^249^ Interleukin-27 (IL-27) and IL-6 activate Hh signaling in CD133^+^ non-small-cell lung CSCs.^250^ During self-renewal and maintenance of stemness of BCMab1^+^CD44^+^ bladder CSCs, glycotransferase GALNT1-mediated glycosylation significantly activates Sonic Hh signaling by upregulating Gli1.^251^

Furthermore, p63, a master regulator of normal epithelial stem cell maintenance, regulates the expression of Shh, Gli2, and PTCH1 by directly binding to their gene regulatory regions, which eventually contributes to the activation of Hh signaling in mammary CSCs.^252^ The N-terminal domain of forkhead box C1 binds directly to an internal region (amino acids (aa) 898–1168) of Gli2 to enhance transcriptional activation of Gli2 and determines the stem cell phenotype in breast CSCs.^253^ Through recruitment of the deubiquitinating enzyme ATXN3, tetraspanin-8 interacts with PTCH1 and inhibits the degradation of the SHH/PTCH1 complex. In addition, long noncoding microRNAs also activate Hh signaling. For example, lncHDAC2 promotes the self-renewal of liver CSCs by recruiting the NuRD complex onto the promoter of the *PTCH1* gene to suppress its expression.^254^ In addition, the TME is crucial for the survival of CSCs. Consequently, breast CSCs secrete Shh, which upregulates cancer-associated fibroblasts (CAFs). Subsequently, CAFs secrete factors that promote the expansion and self-renewal of breast CSCs.^255^ Hh signaling also promotes self-renewal and metastasis of CSCs by upregulating the expression of related downstream markers of CSCs, such as Bmi-1, Wnt2, ALDH1, CD44, CCND1, Twist1, C-MYC, Nanog, Oct4, PDGFRα (platelet-derived factor receptor-α), Snail, Jagged 1, and C-MET.^231,247,256–264^

Some proto-oncogenes and suppressor genes also directly or indirectly regulate Hh signaling in the proliferation and migration of CSCs. The signal peptide CUB EGF-like domain-containing protein 2 (SCUBE2), a member of the SCUBE family of proteins, inhibits cell proliferation and migration in glioma stem cells by downregulating Hh signaling.^265^ BCL6, a transcriptional repressor and lymphoma oncoprotein, directly represses the Sonic Hh effectors Gli1 and Gli2 in medulloblastoma stem cells.^266^ The transcription factor RUNX3 suppresses metastasis and the stemness of colorectal CSCs by promoting ubiquitination of Gli1 at the intracellular level.^267^ Vasohibin 2 suppresses Smo, Gli1, and Gli2 expression in pancreatic CSCs.^268^ β-Catenin stably increases its physical interaction with Gli1, resulting in Gli1 degradation in medulloblastoma stem cells.^269^ In addition, microRNAs also target Hh signaling components to regulate CSC proliferation. For example, miR-324-5p significantly decreases SMO and Gli1 in myeloma stem cells.^270^ Mir-326 directly downregulates SMO and Gli2 in medulloblastoma stem cells.^271^ MiR-326 downregulates SMO in glioma stem cells.^272^ Mir-122 targets Shh and Gli1 in lung CSCs.^273^ These data demonstrate that amplified Hh signaling is important for the self-renewal, growth, and metastasis of CSCs.

#### NF-κB signaling pathway in CSCs

Nuclear factor-κB (NF-κB), a rapidly inducible transcription factor,^274^ consists of five different proteins (p65, RelB, c-Rel, NF-κB1, and NF-κB2). The main physiological function of NF-κB is the p50-p65 dimer.^275–277^ The primary mode of NF-κB regulation occurs at the level of subcellular localization. In the activation stage, transcription factor complexes must translocate from the cytoplasm to the nucleus.^278^ The activity of the complexes is regulated by two major pathways (canonical NF-κB signaling and noncanonical NF-κB signaling). In the canonical NF-κB activation pathway, activation occurs through the binding of ligands, such as bacterial cell components, IL-1β, TNF-α, or lipopolysaccharides, to their respective receptors, such as Toll-like receptors, TNF receptor (TNFR), IL-1 receptor (IL-1R), and antigen receptors.^279^ Stimulation of these receptors leads to the phosphorylation and activation of IκB kinase (IKK) proteins, subsequently initiating the phosphorylation of IκB proteins.^276^ The alternative pathway of NF-κB activation is termed the noncanonical pathway. The noncanonical pathway receptor originates from different classes, such as CD40, receptor activator for NF-κB, B cell activation factor, TNFR2 and Fn14, and lymphotoxin β-receptor.^280^ This pathway leads to activation of NF-κB by inducing the kinase (NIK), which then phosphorylates and predominantly activates IKK1. The activity of the latter enzyme induces the phosphorylation of p100 to generate p52.^281^

The NF-κB pathway plays an important role in regulating immune and inflammatory responses. In addition, the NF-κB pathway is involved in cellular survival, proliferation, and differentiation.^276^ The process of tumor development and progression produces cytokines, growth, and angiogenic factors and proteases to activate NF‐κB signaling.^282^ Inflammation has been recognized as a hallmark of cancer.^283^ Overactivation of NF-κB signaling has been reported in gastrointestinal, genitourinary, gynecological, and head and neck cancers, breast tumors, multiple myeloma, and blood cancers.^278,284–286^ However, direct or altered molecular mutations in NF-κB have rarely been reported in human cancers.^287^ Based on recent studies, NF-κB regulates many genes and is implicated in cell survival, proliferation, metastasis, and tumorigenesis of cancer.^288^ NF-κB activation also directly or indirectly enhances the expression of key angiogenesis factors and adhesion molecules, such as IL-8, vascular endothelial growth factor (VEGF), and growth-regulated oncogene 1.^289^

**Fig. 3:**
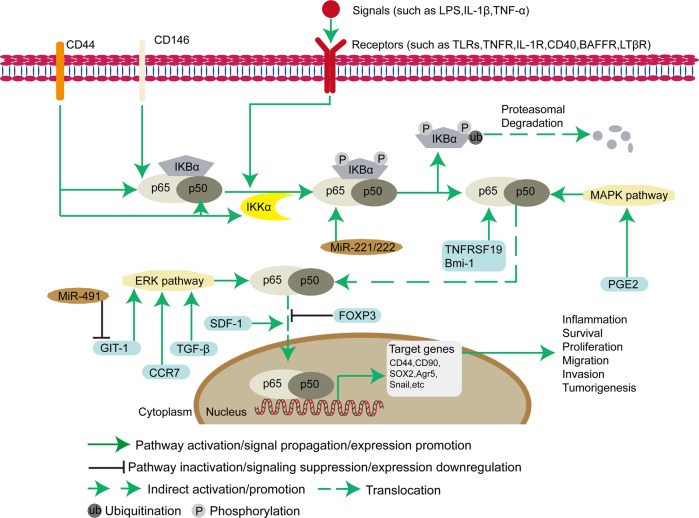
NF-κB signaling pathway in cancer stem cells NF-κB proteins are involved in the dimerization of transcription factors, regulate gene expression, and affect various CSC biological processes, including inflammation, stress responses, growth, and development of CSCs. The main physiological function of NF-κB is the p50-p65 dimer. The active p50-p65 dimer is further activated by post-translational modification (phosphorylation, acetylation, or glycosylation) and transported into the nucleus, which induces the expression of target genes in combination with other transcription factors. Some proteins (CD44, CD146, TNFRSF19, Bmi-1, FOXP3, and SDF-1) and microRNAs (miR-221 and miR-222) directly regulate the NF-κB pathway. In addition, some proteins (PGE2, GIT-1 (G protein-coupled receptor kinase-interacting protein 1), C-C chemokine receptor 7 (CCR7), and TGF-β) and miR-491 indirectly affect the NF-κB pathway via the ERK and MAPK pathways in CSCs

The NF-κB pathway has an essential connection regulating inflammation, self-renewal, or maintenance and metastasis of CSCs (Fig. 3). CD44^+^ cells promote self-renewal, metastasis, and maintenance of ovarian CSCs by increasing the expression of RelA, RelB, and IKKα and mediating nuclear activation of p50/RelA (p50/p65) dimer.^290^ High levels of NIK induce activation of the noncanonical NF-κB pathway to regulate the self-renewal and metastasis of breast CSCs.^291^ Moreover, stromal cell-derived factor-1 (SDF-1) also has the same effect by regulating the translocation of p65 from the cytoplasm to the nucleus.^292^ The inflammatory mediator prostaglandin E2 (PGE2) contributes to tumor formation, maintenance, and metastasis by activating NF-κB via EP4-PI3K (phosphoinositide 3-kinase) and EP4-MAPK pathways in colorectal CSCs.^293^ Chemokines, low-molecular-weight proinflammatory cytokines, are important mediators of cell proliferation, metastasis, and apoptosis.^294^ C-C chemokine receptor 7 interacts with its ligand chemokine ligand 21 to inhibit apoptosis and induce survival and migration in CD133^+^ pancreatic cancer stem-like cells by increasing the expression of extracellular signal-regulated kinase 1/2 (Erk1/2) and p65.^295^ Furthermore, B cell-specific Moloney murine leukemia virus integration site 1 (Bmi-1) also enhances the p65 protein in gastric CSCs.^296^ MicroRNAs also play an important role in promoting the proliferation of CSCs. Mir-221/222 promotes self-renewal, migration, and invasion in breast CSCs by inhibiting the expression of PTEN and then inducing the phosphorylation of AKT, resulting in elevated p65, p-p65, and COX2.^297^

In addition, other transcription factors also inhibit self-renewal and metastasis in CSCs by the NF-κB pathway. Increased expression of FOXP3 has been identified in different cancers.^298^ FOXP3 interacts with NF-κB, inhibits the expression of COX2 located downstream of NF-κB, and affects self-renewal and metastasis in colorectal CSCs.^299^ Overexpression of miR-491 blocks the activation of NF-κB in liver CSCs by targeting G protein-coupled receptor kinase-interacting protein 1, which inhibits ERKs.^300^ Moreover, some drugs inhibit cell proliferation and metastasis of CSCs by the NF-κB pathway. Disulfiram, an anti-alcoholism drug, inhibits tumor growth factor-β (TGF-β)-induced metastasis via the ERK/NF-κB/Snail pathway in breast CSCs.^301^ Sulforaphane preferentially inhibits self-renewal in triple-negative breast CSCs by inhibiting NF-κB p65 subunit translocation and downregulating p52 and its transcriptional activity.^302^ Curcumin regulates the proliferation, metastasis, and apoptosis of HCC stem cells by inhibiting the NF-κB pathway.^303^ These data demonstrate that amplified NF-κB signaling is important for regulating apoptosis, proliferation, and metastasis of CSCs.

#### JAK-STAT signaling pathway

The Janus kinase/signal transducers and activators of transcription (JAK-STAT) signaling pathway is a signal transduction pathway that is stimulated by cytokines. This pathway is involved in many important biological processes, such as cell proliferation, differentiation, apoptosis, and immune regulation. Compared with the complexity of other signaling pathways, this signaling pathway is relatively simple. There are three components: the tyrosine kinase-related receptor, the tyrosine kinase JAK, and the transcription factor STAT.^304^ Many cytokines and growth factors transmit signals through the JAK-STAT signaling pathway, including interleukin-2-7, granulocyte/macrophage colony-stimulating factor, growth hormone, EGF, PDGF, and interferon.^305^ These cytokines and growth factors have corresponding receptors on the cell membrane. The common characteristic of these receptors is that the receptor itself does not have kinase activity, but there is a binding site for the tyrosine kinase JAK in the cells. After binding with ligands, tyrosine residues of various target proteins are phosphorylated through JAK activation to achieve signal transduction from the extracellular to intracellular space. The JAK protein family consists of four members: JAK1, JAK2, JAK3, and Tyk2.^306^ JAK proteins have seven JAK homology (JH) domains in their structures. The JH1 domain is the kinase domain, the JH2 domain is the "pseudo" kinase domain, and JH6 and JH7 are the receptor binding domains.^307^ STAT is called "signal transducer and activator of transcription". As the name implies, STAT plays a key role in signal transduction and transcriptional activation. At present, seven members of the STAT family (STAT1, STAT2, STAT3, STAT4, STAT5a, STAT5b, STAT6) have been identified. The structure of STAT protein can be divided into the following functional regions: N-terminal conserved sequence, DNA-binding region, SH3 domain, SH2 domain, and C-terminal transcriptional activation region.^308^ Generally, many cytokines and growth factors integrate with tyrosine kinase-related receptors. After receiving the signal from the upstream receptor molecule, JAK is quickly recruited to and activates the receptor, resulting in JAK activation to catalyze tyrosine phosphorylation of the receptor. The phosphorylated tyrosine on the receptor molecule, which is a signaling molecule, can bind with the SH2 site of STAT.^309^ When STAT binds to the receptor, tyrosine phosphorylation of STAT also occurs, which forms a dimer and enters the nucleus.^310^ As an active transcription factor, the STAT dimer directly affects the expression of related genes and then changes the proliferation or differentiation of target cells.^311^

Constitutive activation of JAKs and STATs was first recognized as being associated with malignancy in the 1990s.^312^ Based on current studies, JAK2 mutation and abnormal activation of STAT3 are prone to occur in many tumors.^313^ Mutations in JAK2 have been reported in the majority of patients with myeloproliferative neoplasms,^314^ such as polycythemia vera, myelofibrosis, and thrombocythemia.^315,316^ These disorders are caused by the overexpansion of hematopoietic precursors, which are often clonal and can result in leukemia.^314^ Several lines of evidence show that constitutive activation of JAK2 and STAT3 in the absence of any stimulating ligand occurs in polycythemia vera.^317,318^ Moreover, studies have also found aberrant activation of STATs in human cancers, such as head and neck cancer,^319^ endometrial cancer,^320^ breast cancer, diffuse large B cell lymphoma,^321^ HCC,^322^ colorectal cancer, glioma,^323^ and colon cancer.^324^ Furthermore, aberrant STAT5 signaling has been found in the pathogenesis of hematologic and solid organ malignancies.^325,326^

The JAK/STAT pathway is evolutionarily conserved. This pathway promotes the survival, self-renewal, hematopoiesis, and neurogenesis of ESCs.^327^ This pathway is also activated in CSCs. The persistent activation of STAT3 significantly promotes cell survival and the maintenance of stemness in breast CSCs.^328^ IL-10 induces cell self-renewal, migration, and invasion in non-small-cell lung CSCs.^329^ IL-6 activates the JAK1/STAT3 pathway in ALDH^high^ CD126^+^ endometrial CSCs.^320^ Furthermore, IL-6 also induces the conversion of nonstem cancer cells into cancer stem-like cells in breast cancer by the activating downstream *Oct4* gene.^330^ *Oct4* also activates the JAK1/STAT6 pathway in ovarian CSCs.^331^ In CD44^+^CD24^−^ breast and colorectal CSCs, erythropoietin, and IL-6 activate the JAK2/STAT3 pathway.^332–334^ Retinol-binding protein 4 activates JAK2/STAT3 signaling by its STRA6 receptor in colon CSCs.^319^ HIF-1α enhances the self-renewal of glioma stem-like cells by the JAK1/STAT3 pathway.^335^ AJUBA is a scaffold protein that participates in the regulation of cell adhesion, differentiation, proliferation, and migration and promotes the survival and proliferation of colorectal CSCs via the JAK1/STAT1 pathway.^336^

Moreover, microRNAs are also involved in activating JAK/STAT signaling by inhibiting negative regulatory factors of JAK2/STAT3. For example, miR-500a-3p targets multiple negative regulators of the JAK2/STAT3 signaling pathway, such as SOCS2, SOCS4, and PTPN, in HCC stem cells, leading to constitutive activation of STAT3 signaling.^322^ MiR-30 targets SOCS3 in glioma stem cells.^337^ Mir-93 downregulates the expression of JAK1 and STAT3 to induce the differentiation of breast CSCs. Mir-218 negatively regulates the IL-6 receptor and JAK3 gene expression in lung CSCs.^338^ In addition, some endogenous or exogenous genes inhibit JAK/STAT signaling in CSCs. Von Hippel–Lindau suppresses the tumorigenicity and self-renewal ability of glioma stem cells by inhibiting JAK2/STAT3.^323^ Although there are few studies on JAK in CSCs, there is a role for JAK/STAT signaling in the survival, self-renewal, and metastasis of CSCs.

#### TGF/SMAD signaling pathway in CSCs

The TGF-β signaling pathway is involved in many cellular processes associated with organism and embryo development, including cell proliferation, differentiation, apoptosis, and homeostasis. Although the TGF-β signaling pathway regulates a wide range of cellular processes, its structure is relatively simple. TGF-β superfamily ligands bind to a type II receptor, which recruits a type I receptor and phosphorylates it. This type I receptor phosphorylates receptor-regulated Smads (R-Smads), which bind to common pathway Smad (co-Smad). The R-Smad/co-Smad complex acts as a transcription factor and accumulates in the nucleus to regulate the expression of target genes. TGF-β superfamily ligands include BMPs, growth and differentiation factors (GDFs), anti-Mullerian hormone (AMH), activin Nodal, and TGF-β.^339^ These ligands can be divided into two groups, TGF-β/activin and BMP/GDF. The TGF-β/activin group includes TGF-β, activin, and Nodal, and the BMP/GDF group includes BMP, GDF, and AMH ligands.^340^ Based on Smad structure and functions, Smad proteins can be divided into three subfamilies: receptor-activated or pathway-restricted Smad (R-Smads), Co-Smad, and inhibitory Smad (I-Smads), which includes at least nine Smad proteins.^341,342^ R-Smads are activated by type I receptors and form transient complexes with these receptors. There are two types of Smad complexes: AR-Smads are activated by activin TGF-β, including Smad2 and Smad3, and BR-Smads are activated by BMP, including Smad1, Smad5, Smad8, and Smad9. Co-Smad, including Smad4, is a common medium in various TGF-β signal transduction processes. I-Smads, including Smad6 and Smad7, bind to activated type I receptors and inhibit or regulate signal transduction of the TGF-β family.^343^

Many studies have shown that activation of TGF/Smad signaling also occurs in human cancers. Dkk-3, a secreted protein, inhibits TGF-β-induced expression of matrix metallopeptidase 9 (MMP9) and MMP13 to prevent migration and invasion of prostate cancer.^344^ Cancer upregulated gene 2 promotes cellular transformation and stemness, which is mediated by nuclear NPM1 protein and TGF-β signaling in lung cancer.^345^ TGF/Smad also plays an important role in the cell proliferation of CSCs. Cyclin D1 interacts with and activates Smad2/3 and Smad4, promoting cyclin D1-Smad2/3-Smad4 signaling to regulate self-renewal of liver CSCs.^346^ CD51 binds to TGF-β receptors to upregulate TGF-β/Smad signaling in colorectal CSCs.^341^ Upregulation of TGF-β1 induces the expression of smad4, p-Smad2/3, and CD133 in liver CSCs.^347^ TGF-β1 also upregulates the expression of PFKFB3 through activation of the p38 MAPK and PI3K/Akt signaling pathways to regulate glycolysis in glioma stem cells.^348^ Furthermore, silencing ShcA expression also induces activation of STAT4 in breast CSCs.^349^ Moreover, miR-148a inhibits the TGF-β/Smad2 signaling pathway in HCC stem cells.^350^ Smad7, a newly discovered target gene of miR-106b, is an inhibitor of TGF-β/Smad signaling, which inhibits sphere formation of gastric cancer stem-like cells.^351^ Although there are few studies on the TGF/Smad signaling pathway in CSCs, this pathway still plays a very important role.

#### PI3K/AKT/mTOR signaling pathway in CSCs

Phosphatidylinositol-3-kinase (PI3K) is an intracellular phosphatidylinositol kinase.^352^ It consists of the regulatory subunit p85 and catalytic subunit p110, which have serine/threonine (Ser/Thr) kinase and phosphatidylinositol kinase activities.^353^ AKT is a serine/threonine kinase that is expressed as three isoforms: AKT1, AKT2, and AKT3.^354^ AKT proteins are crucial effectors of PI3K and are directly activated in response to PI3K. One of the key downstream target genes of AKT is the mammalian target of rapamycin (mTOR) complex, which is a conserved serine/threonine kinase. It forms two distinct multiprotein complexes: mTORC1 and mTORC2.^355^ mTORC1 consists of mTOR, raptor, mLST8, and two negative regulators, PRAS40 and DEPTOR.^356,357^ mTORC2 phosphorylates AKT at serine residue 473, which leads to full AKT activation.^358^

Studies show that mutations in PTEN lead to the inhibition of PI3K/mTOR signaling in glioblastoma multiforme. However, deletion of PTEN in neural stem cells leads to a neoplastic phenotype that includes cell growth promotion, resistance to cell apoptosis, and increased migratory and invasive properties in vivo.^359^ Inactivation of PTEN and activation of protein kinase B have been found in other solid tumors, such as myeloproliferative neoplasia and leukemia.^360^ Therefore, the PI3K/mTOR signaling pathway is vital for cell proliferation and survival. Abnormal activation of PI3K/mTOR signaling is found in some cancers, such as non-small-cell lung cancer,^361^ breast cancer,^362^ prostate cancer,^363^ Burkitt lymphoma,^364^ esophageal adenocarcinoma,^365^ and colorectal cancer.^366^

Although PI3K/AKT/mTOR has been extensively studied in cancers, there are few studies in CSCs.^358^ PI3K/Akt/mTOR signaling is involved in ovarian cancer cell proliferation and the epithelial–mesenchymal transition.^367^ This signaling activation also enhances the migration and invasion of prostate and pancreatic CSCs.^368,369^ Downregulation of PTEN induces PI3K activation to promote survival, maintenance of stemness, and tumorigenicity of CD133^+^/CD44^+^ prostate cancer stem-like cell populations.^370^ PI3K activation promotes cell proliferation, migration, and invasion in ALDH^+^CD44^high^ head and neck squamous CSCs.^371^ Activation of mTOR promotes the survival and proliferation of breast CSCs and nasopharyngeal carcinoma stem cells.^328,372^ mTORC1 activation also increases aldehyde dehydrogenase 1 (ALDH1) activity in colorectal CSCs.^373^ Activation of mTORC2 upregulates the expression of the hepatic CSC marker EpCAM (epithelial cellular adhesion molecule) and tumorigenicity in hepatocellular CSCs.^374^ Nucleotide-binding domain and leucine-rich repeats (NLRs) belong to a large family of cytoplasmic sensors. NLRC3 (also known as CLR16.2 or NOD3) is associated with PI3Ks and blocks activation of PI3K-dependent kinase AKT in colorectal CSCs.^375^

In addition, some studies have shown that the mTOR signaling pathway is closely related to the metabolism of CSCs. For example, low folate (LF) stress reprograms metabolic signals through the activated mTOR signaling pathway, promoting the metastasis and tumorigenicity of lung cancer stem-like cells.^376^ However, matcha green tea (MGT), an inhibitor of mTOR, inhibits the proliferation of breast CSCs by targeting mitochondrial metabolism, glycolysis, and multiple cell signaling pathways.^377^ A link between the PI3K/Akt/mTOR pathway and CSCs is clearly evident.

#### PPAR signaling pathways in CSCs

Peroxisome proliferator-activated receptors (PPARs) are ligand-activated nuclear transcription factors that were first cloned from mouse liver by Isseman and Green.^378^ PPARs are also members of the ligand-activated transcription factor superfamily of nuclear hormone receptors that are associated with retinoic acid, steroids and thyroid hormone receptors. PPARs act as fat sensors to regulate the transcription of lipid metabolic enzymes.^379^ At present, three subtypes, PPARα, PPARβ, and PPARγ (encoded by the *PPARA*, *PPARD*, and *PPARG* genes, respectively), have been found.^380^ PPARα is highly expressed in hepatocytes, cardiac myocytes, intestinal cells, and renal proximal convoluted tubule cells. PPARγ is abundantly expressed in adipose tissue, vascular parietal cells (such as monocytes/macrophages, ECs, and smooth muscle cells), and myocardial cells.^381^ PPARβ is expressed in almost all tissues of the body, and its expression level is higher than that of PPARα or PPARγ.^382^ In recent years, studies have found that PPARs are closely related to energy (lipid and sugar) metabolism, cell differentiation, proliferation, apoptosis, and inflammatory reactions.^383^ PPARs can exert positive or negative effects to regulate target gene expression by binding to a specific peroxisome located at each gene regulatory site and a proliferative response element.^378^ Their natural ligands are unsaturated fatty acids, eicosane acids, oxidized low-density lipoprotein, very low-density lipoprotein, and linoleic acid derivatives.^384^

To date, there have been many reports about the role of PPARs in cancer cells, including prostate cancer, breast cancer, glioblastoma, neuroblastoma, pancreatic cancer, hepatic cancer, leukemia, and bladder cancer and thyroid tumors.^385^ However, the function of PPARs in CSCs is not well understood, except for some reports on PPARγ. As a tumor suppressor, PPARγ binds and activates a canonical response element in the *miR-15a* gene in breast CSCs to reduce the CD49^high^/CD24^+^ mesenchymal stem cell (MSC) population and inhibit angiogenesis.^386^ PPARγ activation also prevents cell spheroid formation and stem cell-like properties in bladder CSCs and induces adipocyte differentiation and β-catenin degradation in adipose tissues.^387^ Furthermore, expression of PPARγ restrains YAP transcriptional activity to induce differentiation in osteosarcoma stem cells^388^ and melanoma cells.^389^ The PPARγ/NF-κB pathway promotes M2 polarization of macrophages to prevent cell death in ovarian CSCs^4.^^390^ PPARγ activation promotes expression of its target gene *PTEN* to inhibit PI3K/Akt/mTOR signaling, which stunts self-renewal, tumorigenicity, and metastasis in cervical CSCs, glioblastoma stem cells, and liver CSCs.^391,392^ However, combined expression of Dnmt3a and Dnmt3b inhibits PPARγ expression by direct methylation of its promoter in squamous carcinomas.^393^ PPARs are also closely related to the metabolism of CSCs. PPARα and PPARβ/δ regulate metabolic reprogramming in glioblastoma stem cells, lung CSCs, and mouse mammary gland cancer.^394^ The transcription coactivator peroxisome proliferator-activated receptor gamma coactivator 1α (PPARGC1A, also known as PGC-1α) promotes CSC proliferation and invasion by enhancing oxidative phosphorylation, mitochondrial biogenesis, and the oxygen consumption rate of breast CSCs.^395^ In addition, the AMPK signaling pathway (adenosine 5′-monophosphate (AMP)-activated protein kinase) is an AMP-dependent protein kinase that is a key molecule in the regulation of bioenergy metabolism and is the core of the study of diabetes and other metabolic-related diseases. AMPK is expressed in various CSCs related to metabolism. Some studies have shown that AMPK is necessary for prostate CSCs to maintain glucose balance.^396^ Metformin, an antidiabetic drug that fights cancer, targets AMPK signaling to inhibit cell proliferation and metabolism in colorectal CSCs^397^ and HCC stem cells.^398^ Therefore, metformin may be a potential therapeutic regent by regulating the energy metabolism of CSCs. These studies suggest that PPARs play an important role in the growth of CSCs.

#### Interactions between signaling pathways in CSCs

As mentioned previously, these complex signal transduction pathways are not linear. In some cases, crosstalk between and among various pathways occurs to regulate CSCs.^399^ Wnt/β-catenin and NF-κB signaling work together to promote cell survival and proliferation of CSCs. TNFRSF19, a member of the TNF receptor superfamily, is regulated in a β-catenin-dependent manner, but its receptor molecules activate NF-κB signaling to regulate the development of colorectal cancer.^400^ Knockdown of CD146 results in inhibition of NF-κB/p65-initiated GSK3β expression, which promotes nuclear translocation and activation of β-catenin.^401^ In addition, there is negative regulation between Wnt/β-catenin and NF-κB signaling. Studies have revealed a negative effect of β-catenin on NF-κB activity in liver, breast, and colon cancer cells.^402,403^ Leucine zipper tumor suppressor 2 (LZTS2) is a putative tumor suppressor, and NF-κB activation inhibits β-catenin/TCF activity through upregulation of LZTS2 in liver, colon, and breast cancer cells.^404–406^ Wnt/β-catenin and Hh signaling have important functions in embryogenesis, stem cell maintenance, and tumorigenesis. Wnt/β-catenin signaling induces the expression of CRD-BP, an RNA-binding protein, which results in the binding and stabilization of Gli1 mRNA, leading to an increase in Gli1 expression and transcriptional activity, which promotes the survival and proliferation of colorectal CSCs.^407^ However, a report showed that noncanonical Hh signaling is a positive regulator of Wnt signaling in colon CSCs.^408^

In addition, crosstalk between pathways promotes cell growth and metastasis through maintenance of the CSC population. Downregulation of Notch1 and IKKα enhances NF-κB activation to promote the CD133^+^ cell population in melanoma CSCs.^409^ IL-6/JAK/STAT3 and TGF-β/Smad signaling induce the proliferation and metastasis of lung CSCs.^410^ IL-17E binding to IL-17RB activates the NF-κB and JAK/STAT3 pathways to promote proliferation and sustain self-renewal of CSCs in HCC.^411^ TGF-β1 silencing decreases the expression of Smad2/3, β-catenin, and cleaved-Notch1 to inhibit the activation of Wnt and Notch signaling in liver CSCs.^346^ Activation of TGF-β1 induces lncRNA NKILA expression to block NF-κB signaling, which inhibits metastasis of breast CSCs.^412^ TGF-β also directly regulates the expression of Wnt5a in breast CSCs to limit the stem cell population.^413^ Furthermore, Notch, IKK/NF-κB, and other pathways together regulate the proliferation and metastasis of CD133^+^ cutaneous SCC stem cells.^409^ PI3K/mTOR signaling upregulates the expression of STAT3 to promote the survival and proliferation of breast CSCs.^328^ Inhibition of TORC1/2 increases FGF1 and Notch1 expression. The PI3K/AKT/mTOR and Sonic Hh pathways cooperate to inhibit the growth of pancreatic CSCs.^414^ Increasing evidence shows that crosstalk regulates the survival, self-renewal, and metastasis of CSCs.

### The microenvironment of CSCs

**Fig. 4:**
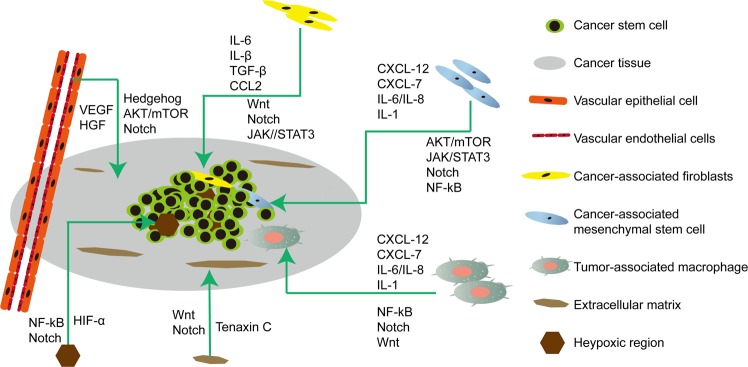
The microenvironment of cancer stem cells. Proliferation, self-renewal, differentiation, metastasis, and tumorigenesis of CSCs in the CSC microenvironment. The CSC microenvironment is mainly composed of vascular niches, hypoxia, tumor-associated macrophages, cancer-associated fibroblasts, cancer-associated mesenchymal stem cells, and extracellular matrix. These cells in response to hypoxic stress and matrix induce growth factors and cytokines (such as IL-6 and VEGF) to regulate the growth of CSCs via Wnt, Notch, and other signaling pathways

CSCs interact with the microenvironment through adhesion molecules and paracrine factors. The microenvironment provides a suitable space for the self-renewal and differentiation of CSCs, protects CSCs from genotoxicity, and increases their chemical and radiological tolerance. The TME mainly consists of the tumor stroma, adjacent tissue cells, microvessels, immune cells, and immune molecules.^415^ CSCs not only adapt to changes in the TME but also affect the TME. Concurrently, the microenvironment also promotes the self-renewal of CSCs, induces angiogenesis, recruits immune and stromal cells, and promotes tumor invasion and metastasis (Fig. 4).

#### Vascular niche microenvironments and CSCs

The normal vasculature is composed of ECs, basement membranes, and parietal cells. ECs are the basis for the formation of the inner surface of blood vessels.^416^ Studies reported that glioblastoma stem cells are located around the blood vessels, and the concept of the cancer microvascular environment was first proposed. Calabrese et al.^417^ demonstrated that direct contact between ECs and CSCs occurs in brain tumors. CSCs are also found near ECs in other cancers, such as papilloma and colorectal cancer.^418,419^ A study also showed that CD133^+^/CD144^−^ glioma stem cell-like cells differentiate into cancer cells and endothelial progenitor cells and finally into mature ECs.^420^ CSCs differentiate into cancer vascular stem cells/progenitor cells and are directly involved in angiogenesis or form vasculogenic mimicry that is directly involved in the microcirculation of tumors.^421,422^ ECs also promote CSC-like transformation and cell growth through Shh activation of Hh signaling.^423^ Moreover, secreted microvesicles of CSCs promote the proliferation of human umbilical vein ECs and form a tube-like structure in vitro and in vivo in mice.^424–426^ This CSC plasticity has also been demonstrated in other tumors, including neuroblastoma, renal, breast, and ovarian cancer.^427–430^

The vascular microenvironment maintains the initial undifferentiated dormancy of stem cells, supports self-renewal, invasion and metastasis of CSCs, and protects CSCs from any injury.^431^ The role of the EC signaling system has been proven in maintaining the survival and self-renewal of head and neck SC stem cells.^432^ Pasquier and colleagues^433^ showed that treatment with EC microparticles in breast and ovarian cancer models increased the number of CSCs and promoted sphere formation of CSCs. The interaction between CSCs and blood vessels promotes the self-renewal of CSCs through the VEGF-Nrp1 loop.^418^ CSCs promote cancer angiogenesis by inducing secretion of the cytokines VEGF and hepatocyte growth factor (HGF) from ECs.^434^ VEGF receptor 2 plays a key role in vasculogenic mimicry formation, neovascularization, and tumor initiation of glioma stem-like cells.^435^ As a result, the secretion of VEGF in stem cell-like glioma cells is higher than that in normal cancer cells^424^ and regulates the proliferation of glioma stem cells through the mTOR signaling pathway.^436^ Subsequent studies have further shown that multiple signals, such as integrin, Notch, and growth factor receptors, are linked to each other on the cell surface to maintain the stemness of CSCs.^437,438^

#### The hypoxia microenvironment and CSCs

Hypoxia is a key component for CSC formation and maintenance.^439^ The hypoxic microenvironment maintains the undifferentiated state of cancer cells, enhances their cloning rate, and induces the expression of CD133 as a specific biomarker of CSCs.^440^ HIFs are important transcription factors that regulate cellular hypoxia responsiveness^441^ and inhibit cell apoptosis.^442^ As a heterodimer, HIF is composed of HIFα and HIFβ.^443^ HIF-1α regulates the proliferation and fate of CSCs in medulloblastoma and glioblastoma multiforme^444^ and activates the NF-κB pathway to promote CSC survival and tumorigenesis.^445^ HIF-2α maintains the survival and phenotype of CSCs.^446^ HIFα also regulates the expression of the target genes GLUT1, GLUT3, LDHA, and PDK1. Thus, CSCs can adapt to a new method of cell energy metabolism and avoid apoptosis caused by hypoxia.^447^

HIFs also regulate the stemness of CSCs. Previous studies have shown that CSCs need to activate HIF-1α and HIF-2α to maintain their self-sustainability under hypoxic conditions^448^ and obtain pluripotency by upregulating the Sox2 and Oct4 genes.^440^ More importantly, activation of C-MYC by HIF-2α is necessary to ensure undifferentiated CSCs.^449^ The Wnt and Notch signaling pathways regulated by hypoxia and can induce the EMT, which promotes the stemness of CSCs and increases the invasiveness and resistance to radiotherapy and chemotherapy.^450^ HIF-1α binds the Notch ICD and enhances its transcriptional activity. In the hypoxic microenvironment of glioma, both HIF-1α and HIF-2α require the Notch signaling pathway to ensure the self-renewal and undifferentiated status of CSCs.^451^

#### Tumor-associated macrophages and CSCs

Macrophages are an important component of the innate immune response and are a group of cells with plasticity and heterogeneity.^452^ Infiltrating and inflammatory macrophages originate from the precursors of bone marrow mononuclear cells.^453^ These precursor cells infiltrate various tissues from blood vessels and differentiate into different subtypes in different microenvironments. There are two subtypes of macrophages: the M1 and M2 phenotypes. The M1 phenotype has anti-inflammatory and anti-tumor effects and secretes proinflammatory factors such as interleukin-1 (IL-1), IL-12, IL-23, TNF-α, chemokine (C-X-C motif) ligand 5 (CXCL5), CXCL9, and CXCL10. M2 macrophages are generally considered to be the phenotype of tumor-associated macrophages (TAMs),^454–456^ have immunosuppressive and angiogenesis-promoting effects, and are considered to be a tumor-promoting cell type.^456,457^ M2 macrophages secrete CCL17 (C-C chemokine ligand 17), CCL22, and CCL24 and have low expression of IL-12 and high expression of IL-10. Cytokines secreted by macrophages affect the proliferation, tumorigenic transformation, or apoptosis of CSCs through various signaling pathways.^458^

TAMs are closely related to CSCs or stem cell transformation. Renal epithelial cells cocultured with macrophages induce the EMT to transform renal cancer cells into CSCs expressing CD117, Nanog, and CD133.^459^ Another study also showed that mucin-1 secreted by M2 macrophages induces the transdifferentiation of non-small-cell lung cancer cells into CSCs that express CD133 and Sox2.^460^ Jinushi and colleagues^461^ also reported that TAMs secrete MFG-E8, which maintains the self-renewal ability of colon and breast CSCs, and knockout of MFG-E8 significantly inhibits the tumorigenic ability in SCID mice.^461^ TAMs are closely related to glioma stem cell growth.^462^ TAMs are mainly distributed near CD133^+^ glioma stem cells and accumulate in pericapillary and hypoxic areas.^463^ Glioma stem cells recruit and maintain macrophages by secreting a potent chemokine membrane protein.^464^ The ablation of TAMs inhibits the tumorigenesis of glioma stem cells.^465^ Recent studies have shown that the interaction between the TME and CSCs is regulated by a variety of signaling pathways.^466^ Macrophages enhance the invasion of glioma stem-like cells through the TGF-β1 signaling pathway.^467^ TAMs activate the STAT3/Sox2 signaling pathway in mouse breast CSCs by secreting EGF, which promotes the self-renewal ability of CSCs.^468^ IL-8 secreted by TAMs also induces the EMT in hepatocellular cancer cells by activating the JAK2/STAT3/Snail pathway.^469^

#### Cancer-associated fibroblasts and CSCs

CAFs are one of the most important components of the TME and are critical in tumor development and metastasis.^470^ The origin of these cells in the stroma is not entirely clear. Current studies hypothesize that there are five possible sources: (1) transference of fibroblasts in the host stroma;^471^ (2) EMT;^472^ (3) transdifferentiation of perivascular cells;^473^ (4) EMT;^474^ and (5) differentiation of MSCs derived from bone marrow.^475^ In addition, CAFs are also derived from other cell types, such as smooth muscle cells, pericytes, adipocytes, and immune cells.^476^ It is not clear whether there are differences in the functions of CAFs from different sources. CAFs affect cancer cell growth through cell–cell interactions and the secretion of various invasive molecules, such as cytokines, chemokines, and inflammatory mediators.^477–479^

CAFs in the TME play an indispensable role in the generation and maintenance of CSCs.^480^ CAFs transform cancer cells into CSCs.^481^ Studies have shown that CAFs promote the EMT and enhance the expression of prostate CSC markers^482^ by secreting IL-6 and IL-1β in breast cancer.^483,484^ CAFs also secrete TGF-β and activate related pathways to increase ZEB1 transcription, which stimulate lung cancer cells to undergo EMT and CSC transformation.^485^ CAFs secrete matrix metalloproteinases, which induce the EMT and promote the growth of stem cell-specific components in tumors.^482^ Paracrine interaction between CAFs and CSCs is critical for maintaining the CSC niche of lung CSCs.^486^ Fibroblast-derived CCL-2 regulates CSCs through gap activation, thus promoting the progression of tumors.^487^ CAFs and adipocytes also secrete leptin, which increases the globulation rate of breast CSCs in vitro.^488^

CAFs also regulate the proliferation of CSCs by other signaling pathways. For example, CAFs increase the secretion of CCL-2 to activate the Notch1/STAT3 pathway, which increases the expression of stem cell markers and upregulates the globulation rate in breast cancer.^489^ CAFs regulate TIC plasticity in HCC through c-Met/FRA1/HEY1 signaling.^490^ CAFs secrete high levels of IL-6 to activate Notch signaling through STAT3 Tyr705 phosphorylation, thus promoting the stem cell-like characteristics of HCC cells.^491^ Similar studies have shown that CAF-derived exons enhance colon stem cell resistance to 5-fluorouracil by activating the Wnt signaling pathway.^492^

#### Cancer-associated MSCs and CSCs

MSCs have high self-renewal ability and multidirectional differentiation potential.^493^ MSCs also specifically migrate to the injured site and tumor tissue and are easy to isolate and expand in vitro.^494,495^ MSCs are considered to be an ideal vector for gene therapy because of their characteristics of homing to and secreting cytokines in tumors.^496^ However, these tumorigenic characteristics of MSCs still need to be studied. MSCs not only promote tumor development^497,498^ but also inhibit cancer cell growth.^499^ Bone marrow MSCs promote tumor growth by promoting angiogenesis, metastasis, and the survival of CSCs.^500^ MSCs in the TME are conducive to the proliferation, carcinogenesis, and metastasis of breast CSCs through ionic purinergic signal transduction.^501^ MSCs can differentiate into CAFs, and CAFs further regulate CSCs and promote the occurrence and metastasis of cancers.^502^ The possible mechanism is related to the spontaneous fusion between cancer cells and MSCs.^503^ The fusion of MSCs with breast cancer, ovarian cancer, gastric cancer, and lung cancer cells in vitro and in vivo has been confirmed.^504,505^ MSCs regulate the TME by secreting IL-6 to maintain the undifferentiated state of osteosarcoma cells.^506,507^ IL-1 stimulates the secretion of PGE2 via autocrine signaling, which ultimately activates β-catenin signaling in cancer cells in a paracrine manner and transforms cancer cells into CSCs.^508^ In the ECM, bone mesenchymal stem cells activate the NF-κB pathway and induce a CSC phenotype by secreting a variety of cytokines and chemokines, such as CXCL12, CXCL7, and IL-6/IL-8.^509^ The interaction between MDSCs and CSCs via IL-6/STAT3 and Notch signaling is critical to the progression of breast cancer.^510^

#### Extracellular matrix and CSCs

The ECM is an insoluble structural component of the matrix in mesenchymal and epithelial vessels. The ECM includes collagen, elastin, aminoglycan, proteoglycan, and noncollagen glycoprotein.^511,512^ At present, increasing evidence shows that the ECM is an integral part of stem cell niches that regulates the balance of stem cells in three different biological states: static, self-renewal, and differentiation.^513^ Experiments in vitro and in vivo have shown that ECM receptors can be used to aggregate CSCs^514^ and induce drug resistance.^513,515^ Fibronectin, vimentin, collagen, and proteoglycan in the ECM bind to cytokines such as FGF, HGF, VGF, BMP, and TGF-β in the TME and regulate their activities.^516^ In HCC, an increased matrix promotes cell proliferation and chemotherapeutic resistance and increases the expression of CSC-related markers, including CD44, CD133, c-kit, cxcr4, Oct4, and Nanog. Hyaluronic acid in the ECM is a ligand for the CD44 receptor and can regulate the acquisition and maintenance of CSC stemness during mutual contact.^517^ The ECM also binds the Wnt ligand Wnt5b via molecular MMP3 and leads to the expansion and proliferation of mammary epithelial stem cells.^518^ In addition, tenascin C in the ECM maintains the stability of breast CSCs by increasing the activity of the Wnt and Notch signaling pathways.^519^

#### Exosomes in the TME and CSCs

Exosomes are nanovesicles secreted by various types of living cells (30–100 nm in diameter)^520^ and are widely distributed in peripheral blood, saliva, urine, ascites, pleural effusion, breast milk, and other body fluids.^521^ Exosomes contain a large number of functional proteins, RNA, microRNAs, DNA fragments, and other bioactive substances.^522–525^ These bioactive substances mediate material transport and information exchange between cells, thus affecting the physiological function of cells.^526,527^ The exosomes secreted by cancer cells promote angiogenesis,^528^ induce differentiation of tumor-related fibroblasts,^529^ participate in immune regulation of the TME,^530^ and regulate the microenvironment before metastasis.^531^ Clinical analysis has revealed that exosomes are released at higher levels in cancer cells.^532^

Recent studies have shown that endocytosis of lipid rafts in MSCs is associated with increased secretion of exosomes.^533^ Exosome signaling mediates the interaction of CSCs and normal stem cells, thereby regulating oncogenesis and tumor development.^534^ Exosomes also regulate CSC growth by targeting specific signaling pathways, such as Wnt, Notch, Hippo, Hh, and NF-κB.^535–537^ Extracellular vesicles released by glioblastoma stem cells promote neurosphere formation, endothelial tube formation, and the invasion of glioblastoma.^538^ CSCs promote cell proliferation and self-renewal through crosstalk between exosome signal transduction and the surrounding microenvironment.^539^ The exosomes released from CSCs affect signal transduction in nearby breast cancer cells^540^ and increase the stemness of breast cancer cells.^540^ Similarly, fibroblast-derived exosomes contribute to chemoresistance by promoting colorectal CSC growth.^491^ Exosomes in the TME promote the transformation of non-CSCs into CSCs. CAF-derived exosomes significantly increase the ability to form mammary globules and promote the stemness of breast cancer cells.^541^ Similarly, CAF-derived exosomes also promote sphere formation of colorectal cancer cells by activating Wnt signaling and ultimately increase the percentage of CSCs.^491^ Exosomes from glioma-associated MSCs increase the tumorigenicity of glioma stem-like cells by transferring miR-1587.^542^ In addition, exosomes regenerate stem cell phenotypes by mediating the EMT or regulating stem cell-related signaling pathways, such as the Wnt pathway, Notch pathway, Hh pathway and other pathways, which convert cells into CSCs.^543^ Exosomes have many advantages, such as low immunogenicity, biocompatibility, easy production, cytotoxicity, easy storage, high drug loading capacity, and long life and have become ideal drug carriers for cancer therapy.^544–548^

## Therapeutic targeting of CSCs

### Agents targeting CSC-associated surface biomarkers in clinical trials

Monoclonal antibodies (mAbs) that target CSC-specific surface biomarkers have become an emerging technology for cancer therapy. Rituximab, a CD20 mAb, is an active agent for the treatment of follicular lymphoma and mantle-cell lymphoma, but some serious adverse reactions still occur.^549^ Subsequently, to improve the availability and affordability of radioimmunotherapy for refractory or recurrent non-Hodgkin’s lymphoma (NHL), a phase II clinical trial for a radioiodine replacement of rituximab was carried out, which showed a response rate of 71% and a complete remission rate of 54% in 35 patients, with only two cases of grade IV hematologic toxicity observed.^550^ Encouragingly, alemtuzumab, a humanized CD52 antibody, has been approved for the treatment of chronic lymphocytic leukemia (CLL) in patients who failed to respond to alkylating agents and purine. Furthermore, the combination of the CD20 and CD52 antibodies in the treatment of refractory CLL was safe, nontoxic, feasible, and positive.^551^ Another antibody drug, relabeled bivatuzumab, is an anti-CD44v6 mAb,^71^ which was found to be safe when it was used for the treatment of head and neck SCC.^552^ These results have been obtained in subsequent clinical research^553^ and safety/efficacy studies.^554^ Unfortunately, in a stage I dose escalation study with the CD44v6 antibody, one patient with head and neck SCC of the esophagus suffered deadly skin toxicity.^555^

Several CD123 antibodies have been developed, XmAb14045 and MGD006, and were designed with biospecific effects against CD3 and CD123. Talacotuzumab is also effective against CD16 and CD123. CSL360, another CD123 antibody, was used to treat relapsed, refractory, or high-risk acute myeloid leukemia (AML) and displayed no anti-leukemic activity in most cases.^556^ SL-401, another CD123 antibody, was used to treat blastic plasmacytoid dendritic cell neoplasm patients. The results showed major positive responses in seven out of nine patients, including five complete responses and two partial responses.^557^ An ongoing phase II study of SL-401 in 29 patients with blastic plasmacytoid dendritic cell neoplasms demonstrated robust single-agent activity with an 86% overall response rate.^558^ The latest antibodies against CSC surface markers, such as XmAb14045 (NCT02730312), flotetuzumab (NCT02152956), and talacotuzumab (NCT02472145), are also in clinical study. Furthermore, several other markers that can distinguish LSCs from other cells are under clinical development, such as IL-1 receptor accessory protein, CD27/70, CD33, CD38, CD138, CD93, CD99, C-type lectin-like molecule-1, and T cell immunoglobulin mucin-3.

EpCAM, a common CSC biomarker, has also received attention in clinical trials.^559^ Adecatumumab, an EpCAM antibody, was used in patients with hormone-resistant prostate cancer, and the results showed that the EpCAM-specific antibody has great clinical potential.^560^ Catumaxomab, a multifunctional mAb against EpCAM, binds and recognizes EpCAM and the T cell antigen CD3 (anti-EpCAM × anti-CD3).^561^ Intraperitoneal injection of catumaxomab to treat EpCAM-positive ovarian cancer and malignant ascites has shown high efficacy in killing cancer cells and reducing the formation of ascites.^562^ Catumaxomab has been used in non-small-cell lung cancer and also had a good survival rate.^561^ However, other types of EpCAM antibodies, such as edrecolomab^563^ and adecatumumab,^564^ have minimal efficacy in colorectal and breast cancers. Combining EpCAM antibodies with chimeric antigen receptor T cell (CAR-T) technology has also been used in various types of cancers in phase I trials, such as NCT02915445, NCT03563326, NCT02729493, and NCT02725125. With a deeper understanding of CSC surface biomarkers, there has been significant progress in developing antibodies targeting CSCs (Table 2). However, CSC surface phenotypes can vary in different patients or different cancers, and different CSC populations with different phenotypes might coexist. CSCs also diverge or evolve into different cancer cells, acquiring distinct phenotypes upon relapse. Therefore, the strategies used in clinical trials should be determined according to the phenotypes of the different cancers. At the same time, combining different surface antibodies with relevant chemotherapy drugs can achieve an ideal therapeutic effect.

**Table 2:** Agents targeting CSC-associated surface markers in ongoing clinical trials

Drug name	Antibody target	Condition	Sample size	Highest status	NCT number	Current status
Surface antigens						
Catumaxomabr (emovab)	EpCAM/CD3	Ovarian cancer	II	44	NCT00189345	Completed
Tagraxofusp SL-401	CD123	Acute myeloid leukemia	I	36	NCT03113643	Recruiting
KHK2823			I	39	NCT02181699	Terminated
Talacotuzumab			III	326	NCT02472145	Completed, has results
SGN-CD123A			I	17	NCT02848248	Terminated
IMGN632			II	155	NCT03386513	Recruiting
XmAb14045	CD123/CD4		II	105	NCT02730312	Recruiting
MGD006	CD123/CD3		II	179	NCT02152956	Recruiting
JNJ-63709178			III	326	NCT02472145	Completed, has results
CSL362	CD124		I	30	NCT01632852	Completed
TTI-621	CD47	Solid tumor	I	260	NCT02663518	Recruiting
Hu5F9-G4		Solid tumor	I	88	NCT02216409	Completed
IBI188		Advanced malignancies	I	42	NCT03763149	Recruiting
CC-90002		Hematologic neoplasms	I	28	NCT02641002	Terminated
AO-176		Solid tumor	I	90	NCT03834948	Recruiting
SRF231		Solid tumor	I	148	NCT03512340	Recruiting
Bivatuzumab mertansine		Metastatic breast cancer	I	24	NCT02254005	Completed
Vadastuximab talirine (SGN-CD33A)	CD33	Acute myelogenous leukemia	I	195	NCT01902329	Completed
IMGN779			I	62	NCT02674763	Completed
Mylotarg (gemtuzumab ozogamicin)		ECG	IV	56	NCT03727750	Recruiting
RO5429083	CD44	Malignant solid tumors	I	65	NCT01358903	Completed
SPL-108		Ovarian cancer	I	18	NCT03078400	Recruiting
Salazosulfapyridine	CD44V4	Non-small-cell lung cancer	I		UMIN000017854	
AMC303	CD44V6	Solid tumor	I	55	NCT03009214	Recruiting
Immune checkpoints						
Ipilimumab	CTLA-4	Non-small-cell lung cancer	II	24	NCT01820754	Completed, has results
Nivolumab	PD-1	Glioblastoma multiforme	II	29	NCT02550249	Completed
Pembrolizumab			II	80	NCT02337491	Completed, has results
Cemiplimab			II	30	NCT04006119	Recruiting
Idarubicin		Acute myeloid leukemia	II	51	NCT01035502	Completed
Sym021		Solid tumor lymphomas	I	102	NCT03311412	Recruiting
Durvalumab		Solid tumors	II	124	NCT02403271	Completed, has results
Atezolizumab	PD-L1	Non-small-cell lung cancer	III	1225	NCT02008227	Completed, has results
Avelumab		Recurrent glioblastoma	II	52	NCT03291314	Completed
Sym023	Tim3	Solid tumor	I	48	NCT03489343	Recruiting
ARGX-110	CD70	Acute myeloid leukemia	II	36	NCT03030612	Active, not recruiting
Varlilumab (CDX-1127)		Solid tumors	II	175	NCT02335918	Completed
Sym022	LAG3	Solid tumor	I	30	NCT03489369	Recruiting
MGD013	CD70/LAG3	Solid tumors	I	255	NCT03219268	Recruiting

### Agents targeting CSC-associated signaling pathways in clinical trials

The signaling pathways that regulate the maintenance and survival of CSCs have become targets for cancer treatment. At present, the main signaling pathways are the Wnt, Notch, and Hh signaling pathways, as well as the TGF-β, JAK-STAT, PI3K, and NF-κB signaling pathways. These pathways often interact with each other during tumor development and in CSCs. Considerable progress has been made in early clinical trials for Notch and Hh pathway inhibitors, while targeting the Wnt pathway has proven to be difficult.^10^

The Notch signaling pathway plays an important role in the maintenance of CSCs^565,566^ and can induce CSC differentiation. Abnormal activity of the Notch signaling pathway has been observed in many cancers, such as leukemia,^567^ glioblastoma,^568,569^ breast cancer,^570^ lung cancer,^571^ ovarian cancer,^572^ pancreatic cancer,^573^ and colon cancer.^574^ At present, there are three major clinical methods used to inhibit Notch signaling, secretase inhibition (γ-secretase inhibitor (GSI)), Notch receptor or ligand antibodies, and combination therapy with other pathways. For example, GSIs have been tested in clinical trials. Among them, MK-0752 (NCT00100152) was the first GSI used to treat T cell acute lymphoblastic leukemia in children in a phase I trial. However, the study was terminated because of poor results.^575^ MK-0752 also had no clinical activity in extracranial solid tumors in subsequent phase II trials. Only one complete response with interdegenerative astrocytoma and SD extension out of 10 patients with different types of glioma was observed.^576^ MK-0752 is well tolerated and shows targeted inhibition in recurrent pediatric central nervous system tumors.^577^ In addition, combining MK-0752 with cisplatin treatment for ovarian cancer,^578,579^ docetaxel treatment for locally advanced or metastatic breast cancer,^569^ and gemcitabine treatment for ductal adenocarcinoma of the pancreas^580^ has shown good efficacy. However, the clinical effect was minimal in patients with advanced solid tumors,^576,581^ including metastatic pancreatic cancer.^582^

In addition, RO4929097, another selective GSI, showed good anti-tumor activity in preclinical and early trials,^583,584^ but was not good for metastatic colorectal cancer,^585^ metastatic pancreatic adenocarcinoma,^586^ or recurrent platinum-resistant ovarian cancer.^587^ Combinations of RO4929097 with gemcitabine,^588^ temsirolimus,^587^ cediranib,^589^ or capecitabine^590^ in advanced solid tumors, as well as with bevacizumab in recurrent high-grade glioma, are well tolerated and have modest clinical benefits. However, NCT01154452, the combination of RO4929097 with vismodegib and vismodegib alone for patients with advanced osteosarcoma, showed no significant difference in a phase Ib trial. The third oral GSI, PF-03084014, had good efficacy in desmoid tumors either in phase I or subsequent phase II studies.^591^ Preliminary evidence of its clinical efficacy was demonstrated in patients with solid tumors,^592^ as well as in patients with recurrent acute T cell lymphoblastic leukemia.^593^ Other selective GSIs, such as BMS-906024 (NCT01292655), BMS-986115 (NCT01986218), CB-103 (NCT03422679), LY3039478 (NCT02836600), and LY900009 (NCT01158404), have also entered the clinical trial stage, and the results still need to be verified.

DLL4 plays a vital role in regulating tumor angiogenesis.^594^ Therefore, targeting DLL4 is another strategy to block Notch signaling, and this is being tested in the clinic. Demcizumab (OMP-21M18), a humanized IgG2 mAb that targets DLL4 and blocks its interactions with Notch receptors, was tested in a phase I dose escalation study with 55 patients with previously treated solid tumors.^595^ The results have shown that demcizumab had good efficacy against solid tumors, but was not good for metastatic pancreatic cancer treatment when combined with gemcitabine and Abraxane (NCT02289898). NCT02259582, a combination of demcizumab with carboplatin and pemetrexed to treat lung cancers (DENALI study), is ongoing in another phase II study.^595^ Enoticumab, another fully human IgG1 antibody against DLL4, has promising activity in phase I clinical trials for advanced solid malignancies.

Activation of Hh signaling has been implicated in a variety of cancers.^596–598^ Activation of Hh signaling in CSCs contributes to CSC stemness, chemoresistance, and metastatic dissemination. The Hh signaling pathway mainly regulates target gene expression via smoothened (SMO)-mediated nuclear transfer of transcription factors. Three oral SMO antagonists, vismodegib (GDC-0449), sonidegib (LDE225), and glasdegib (PF-04449913), have been approved by the Food and Drug Administration (FDA) and show significant activity in locally advanced and metastatic basal cell carcinoma, as well as in AML.^599–601^ Vismodegib was the first proposed Hh pathway inhibitor in cancer research^602^ and is approved by the FDA^603^ for local or advanced metastatic basal cell carcinoma treatment.^599^ Subsequently, phase I and phase II trials targeting recurrent medulloblastoma have shown that the progression-free survival (PFS) of Shh-mb patients treated with vismodegib is longer and more effective than that of non-Shh-mb patients. Vismodegib even has better activity in patients with recurrent Shh-mb but not in patients with recurrent non-Shh-mb.^604,605^ Vismodegib has also been tested in metastatic colorectal cancer,^606^ pancreatic cancer,^607^ chondrosarcoma,^608^ relapsed/refractory NHL, CLL,^609^ and ovarian cancer.^610^ Disappointingly, these treatments with vismodegib have not resulted in better survival.

Sonidegib was the second SMO antagonist approved for the treatment of locally advanced basal cell carcinoma that recurred after surgery or radiotherapy and is not suitable for surgery or radiation therapy.^611^ In addition, the results of a multicenter, randomized, double-blind phase II trial have shown that 200 mg sonidegib for patients with advanced basal cell carcinoma is the most clinically appropriate dose.^600^

In a phase I study of a 3 + 3 dose escalation to treat small-cell lung cancer patients, sonidegib combined with cisplatin and etoposide sustained PFS in patients with Sox2 amplification.^224^ These combinations in a phase II trial for patients with recurrent medulloblastoma resulted in a complete or partial response in 50% of patients^612^ and have been used for other cancer treatments in phase I/II clinical trials, such as NCT02111187 for prostate cancer, NCT02027376 for breast cancer, and NCT02195973 for recurrent ovarian cancer.

Glasdegib was the first Hh pathway inhibitor approved for the treatment of AML in patients older than 75 years or those unable to use intensive induction chemotherapy^601^ and showed good safety and tolerability in a phase I trial for patients with partial hematologic malignancies in Japan.^613^ In a phase II trial, glasdegib combined with cytarabine/daunorubicin had a significant efficacy in patients with AML, chronic myeloid leukemia (CML) or high-risk myelodysplastic syndromes.^614^ Glasdegib combined with low-dose cytarabine (LDAC) is a potential option for AML patients who are not suitable for intensive chemotherapy.^615^ Other selective SMO inhibitors, including taladegib (LY2940680) and saridegib (IPI-926), have also entered clinical trials for other cancers. As single-target agents, these SMO inhibitors have drug resistance problems. To reduce this problem, some novel inhibitors of terminal components of Hh signaling pathway are being developed, such as arsenic trioxide (ATO)^616^ and GANT-61.^617^

The Wnt signaling pathway is associated with tumor development in breast cancer,^618^ ovarian cancer,^619^ esophageal squamous cell cancer,^620^ colon cancer,^621^ prostate cancer,^622^ and lung cancer.^623^ Until now, several drugs aimed at the Wnt signaling pathway have been in clinical trials, while the majority of Wnt inhibitors are in preclinical testing. Clinical data from initial trials have shown that ipafricept (OMP-54f28/FZD8-Fc) is a first-in-class recombinant fusion protein that antagonizes Wnt signaling.^624^ However, its role in patients with desmoid cancers and germ cell cancers is negligible.^625^ NCT02050178, ipafricept combined with ab-paclitaxel and gemcitabine in patients with untreated stage IV pancreatic cancer, NCT02092363, ipafricept combined with paclitaxel and carboplatin in patients with recurrent platinum-sensitive ovarian cancer, and NCT02069145, ipafricept combined with sorafenib in patients with HCC, are currently being investigated. PRI-724, a β-catenin inhibitor, inhibits the interaction between β-catenin and its transcriptional coactivators. Safety and efficacy testing of PRI-724 for patients with advanced myeloid malignancies (NCT01606579) and advanced or metastatic pancreatic cancer (NCT01764477) have been completed in phase I studies. CWP232291, another inhibitor of β-catenin activity, has also been shown to be effective for AML (NCT03055286) in a phase I clinical study and for recurrent or refractory myeloma (NCT02426723) in a phase I/II clinical study.^626^ Other Wnt signaling inhibitors have also been under clinical trial, including LGK974 (NCT02278133), ETC-159 (NCT02521844), and OMP-18R5 (NCT01973309, NCT01957007, and NCT02005315).

In addition, the mitochondrial glycolysis pathway also plays a key role in regulating the proliferation and apoptosis of CSCs. Venetoclax, a BCL-2 inhibitor, was initially approved by the FDA recently and shows good tolerance and activity for AML patients with adverse reactions.^627^ Two arachidonate 5-lipoxygenase inhibitors, VIA-2291 and GSK2190915, might be potent agents for targeting LSCs in CML,^628^ as shown in Table 3.

**Table 3:** Agents targeting CSC-associated signaling pathways and microenvironment in ongoing clinical trials

Drug name	Target	Condition	Phase	Sample size	NCT number	Current status
Hedgehog inhibitors						
Vismodegib (GDC-0449)	Smoothened	Recurrent or refractory medulloblastoma	II	31	NCT00939484	Completed, has results
		Basal cell carcinoma		28	NCT01700049	Completed, has results
		Sarcoma		78	NCT01700049	Completed, has results
		Recurrent small-cell lung carcinoma		168	NCT01700049	Completed, has results
		Metastatic pancreatic cancer		98	NCT01088815	Completed, has results
		Ovarian cancer		104	NCT00739661	Completed, has results
		Metastatic colorectal cancer		199	NCT00636610	Completed, has results
Sonidegib (LDE225)		Basal cell carcinoma	II	10	NCT01350115	Completed, has results
		Relapsed medulloblastoma		20	NCT01708174	Completed, has results
		Acute myeloid leukemia		70	NCT01826214	Completed, has results
		Pancreatic adenocarcinoma		20	NCT01431794	Completed, has results
		Advanced or metastatic hepatocellular carcinoma		9	NCT02151864	Completed
		Recurrent plasma cell myeloma		28	NCT02086552	Active, not recruiting, has results
		Advanced pancreatic cancer		39	NCT01485744	Active, not recruiting
		Advanced breast cancer	I	12	NCT02027376	Completed, has results
Glasdegib		Acute myeloid leukemia	II	255	NCT01546038	Completed, has results
BMS-833923 (XL139)		Solid tumors	II	12	NCT01413906	Completed
		Small-cell lung carcinoma		5	NCT00927875	Completed
		Metastatic gastric, gastroesophageal, esophageal adenocarcinomas		39	NCT00909402	Completed
		Advanced or metastatic basal cell carcinoma		53	NCT00670189	Completed
		Leukemia		70	NCT01357655	Terminated, has results
Taladegib (LY2940680)		Localized esophageal or gastroesophageal junction cancer	II	9	NCT02530437	Active, not recruiting
		Small-cell lung carcinoma		26	NCT01722292	Terminated, has results
LEQ-506		Solid tumors	I	57	NCT01106508	Completed
G-024856		BCC	I			
Patidegib (IPI-926)		Basal cell carcinomas	II	36	NCT02828111	Completed, has results
		Metastatic or locally advanced chondrosarcoma		105	NCT01310816	Completed
		Metastatic pancreatic cancer		122	NCT01130142	Completed
		Recurrent head and neck cancer	I	9	NCT01255800	Completed
Notch inhibitors						
MK-0752	γ-Secretase	Advanced breast cancer	I	103	NCT00106145	Completed
		Pancreatic cancer	I	44	NCT01098344	Completed
		Metastatic breast cancer	I/II	30	NCT00645333	Completed, has results
RO4929097		Recurrent melanoma	II	14	NCT01196416	Completed, has results
		Advanced or metastatic sarcoma		78	NCT01154452	Completed, has results
		Recurrent renal cell carcinoma		12	NCT01141569	Completed, has results
		Advanced solid tumors		20	NCT01131234	Completed
		Recurrent and/or metastatic epithelial ovarian cancer, fallopian tube cancer, or primary peritoneal cancer		45	NCT01175343	Completed, has results
		Metastatic pancreas cancer		18	NCT01232829	Completed, has results
		Recurrent colon cancer		37	NCT01116687	Completed, has results
		Recurrent or refractory non-small-cell lung cancer		7	NCT01070927	Completed
Nirogacestat (PF-03084014)		Metastatic cancer pancreas	II	3	NCT02109445	Terminated, has results
		Fibromatosis	II	17	NCT01981551	Active, not recruiting
		Triple-negative breast neoplasms	II	19	NCT02299635	Terminated, has results
LY900009		Advanced cancer	I	35	NCT01158404	Completed, has results
Crenigacestat (LY3039478)	Pan-Notch	Advanced solid tumor	I	12	NCT02836600	Active, not recruiting
		T cell acute lymphoblastic leukemia, T cell lymphoblastic lymphoma	I/II	36	NCT02518113	Completed, has results
AL101		Adenoid cystic carcinoma	II	36	NCT03691207	Recruiting
CB-103		Advanced or metastatic solid tumors and hematological malignancies	I/II	165	NCT03422679	Recruiting
BMS-906024		Advanced or metastatic solid tumors	I	94	NCT01292655	Completed
		Lymphoblastic leukemia, acute T cell	I	31	NCT01363817	Completed
Demcizumab (OMP-21M18)	DLL4	Pancreatic cancer	II	207	NCT02289898	Completed, has results
		Non-squamous, non-small-cell neoplasm of lung	II	82	NCT02259582	Completed, has results
Brontictuzumab (OMP-52M51)		Adenoid cystic carcinoma	Not applicable	1	NCT02662608	Completed, has results
Enoticumab (MEDI528)		Advanced solid malignancies	I	83	NCT00871559	Completed
MEDI0639		Solid tumors	I	58	NCT01577745	Completed, has results
Wnt inhibitors						
Ipafricept (OMP-54F28)	Wnt receptor	Solid tumors	I	26	NCT01608867	Completed
		Pancreatic cancer	I	26	NCT02050178	Completed
		Ovarian cancer	I	37	NCT02092363	Completed
		Hepatocellular cancer	I	10	NCT02069145	Completed
Vantictumab (OMP-18R5)		Metastatic breast cancer	I	37	NCT01973309	Completed
		Solid tumors	I	35	NCT01345201	Completed
		Pancreatic cancer	I	30	NCT02005315	Completed
PRI-724	β-Catenin/CBP	Colorectal adenocarcinoma	II	0	NCT02413853	Withdrawn
		Acute myeloid leukemia		49	NCT01606579	Completed
		Solid tumors		23	NCT01302405	Terminated
		Advanced pancreatic cancer		20	NCT01764477	Completed
CWP232291		Acute myeloid leukemia	I	69	NCT01398462	Completed
		Multiple myeloma	I	25	NCT02426723	Completed
LGK974	Porcupine	Metastatic colorectal cancer	I	20	NCT02278133	Completed
		Pancreatic cancer	I	170	NCT01351103	Recruiting
ETC-1922159		Solid tumors	I	65	NCT02521844	Active, not recruiting
Other signaling pathways inhibitors						
Galunisertib (LY2157299)	TGF-β	Prostate cancer	II	60	NCT02452008	Recruiting
LY3200882		Colorectal cancer	II	31	NCT04031872	Not yet recruiting
AVID200		Malignant solid tumor	I	36	NCT03834662	Recruiting
Trabedersen (AP 12009)		Pancreatic neoplasms	II	62	NCT00844064	Completed
		Breast cancer		16	NCT01959490	Completed, has results
		Glioblastoma		141	NCT00431561	Completed
Fresolimumab (GC1008)		Non-small-cell lung carcinoma	II	60	NCT02581787	Recruiting
		Metastatic Breast Cancer		23	NCT01401062	Completed, has results
		Carcinoma Renal cell Melanoma		29	NCT00356460	Completed
Vactosertib (TEW-7197)		Advanced-stage solid tumors	I	35	NCT02160106	Completed
NIS793		Breast cancer Lung cancer Hepatocellular cancer	I	220	NCT02947165	Recruiting
Ruxolitinib	JAK	Metastatic breast cancer	III	29	NCT01594216	Completed
		Myeloproliferative neoplasms		309	NCT00952289	Completed, has results
AZD4205		Advanced non-small-cell lung cancer	II	120	NCT03450330	Recruiting
SAR302503		Hematopoietic neoplasm	II	97	NCT01523171	Completed
SB1518	JAK/FLT3	Acute myelogenous leukemia	II	76	NCT00719836	Completed
PI3K inhibitors						
Alpelisib	PI3K	Advanced breast cancer	II	90	NCT03386162	Recruiting
Buparlisib (BKM120)		Triple-negative metastatic breast cancer	II	50	NCT01629615	Completed
BYL719		Advanced or metastatic gastric cancer	I	18	NCT01613950	Completed
SF1126		Advanced or metastatic solid tumors	I	44	NCT00907205	Completed
SAR245409	PI3K and mTOR	Advanced or metastatic solid tumors	I	146	NCT01390818	Completed, has results
EGFR inhibitors						
Bevacizumab	EGFR	Breast cancer	I	75	NCT01190345	Completed
Matuzumab (EMD 72000)		Esophageal cancer	II	72	NCT00215644	Completed, has results
		Non-small-cell lung carcinoma		150	NCT00111839	Completed, has results
Metabolism inhibitors						
Venetoclax (ABT-199)	BCL-2	Acute myelogenous leukemia	II	32	NCT01994837	Completed, has results
Pegzilarginase	Recombinant pegylated arginase	Small-cell lung cancer	II	84	NCT03371979	Active, not recruiting
131I-TLX-101	LAT1	Glioblastoma multiforme	II	44	NCT03849105	Recruiting
Rifampicin	FAS	Advanced solid tumors	I	36	NCT03077607	Completed, has results
TVB-2640		Advanced breast cancer	II	80	NCT03179904	Recruiting
IM156	AMPK	Advanced solid tumor	I	36	NCT03272256	Recruiting
Telaglenastat	Glutaminase	Solid tumors	II	85	NCT03965845	Recruiting
CB-1158	Arginase	Advanced solid tumors	II	5	NCT03361228	Completed
Niche inhibitors						
Plerixafor (Mozobil)	CXCR4	Advanced pancreatic, ovarian, and colorectal cancers	I	26	NCT02179970	Completed
BL-8040		Metastatic pancreatic adenocarcinoma	II	23	NCT02907099	Active, not recruiting
BKT140		Multiple myeloma	II	16	NCT01010880	Completed
BMS-936564		Relapsed/refractory multiple myeloma	I	46	NCT01359657	Completed
BMS-936564		Acute myelogenous leukemia	I	98	NCT01120457	Completed
LY2510924		Solid tumor	I	9	NCT02737072	Terminated, has results
MSX-122		Refractory metastatic or locally advanced solid tumors	I	27	NCT00591682	Suspended
USL311		Advanced solid tumors and relapsed/recurrent Glioblastoma multiforme	II	120	NCT02765165	Recruiting
AMD3100		Acute myeloid leukemia	II	52	NCT00512252	Completed, has results
Reparixin	CXCR1/2	Breast cancer	II	20	NCT01861054	Terminated
Defactinib (VS-6063)	FAK	Non-small-cell lung cancer	II	55	NCT01951690	Completed

Other abnormal signaling pathways have also been found in CSCs, such as TGF-β, JAK-STAT, PI3K, and NF-κB. These signaling pathways are not independent of each other but rather form a complex signaling network. Agents targeting CSC-associated signaling pathways in ongoing clinical trials are listed in Table 3.

### Targeting the CSC microenvironment

The CSC microenvironment contributes to the self-renewal and differentiation of CSCs and regulates CSC fate by providing cues in the form of secreted factors and cell contact. CXCR4 has been found in most cancers, especially in CSCs. The most well-characterized drug-targeting CXCR4 is plerixafor (AMD3100), and this drug is an effective hematopoietic stem cell mobilizer for patients with multiple myeloma and NHL.^629^ AMD3100 treatment for relapsed or refractory AML resulted in 46% of patients with complete remission with or without white count recovery in a phase I/II study.^630^ In addition, plerixafor with high-dose cytarabine and etoposide treatment for children with relapsed or refractory acute leukemia or myelodysplasia syndrome resulted in two patients with complete remission and one patient with incomplete hematologic recovery out of 18 patients in a phase I study.^631^ LY2510924, a small cyclic peptide, is a potent and selective antagonist of CXCR4 and is well tolerated with no serious adverse events in a phase I trial.^632^ However, the combination of LY2510924 with sunitinib for patients with metastatic renal cell carcinoma has no better effect than sunitinib alone in a phase II trial.^633^ The combination of LY2510924 with carboplatin/etoposide for patients with extensive small-cell lung cancer also had no significant effect compared with that of carboplatin/etoposide alone in a phase II study.^634^ The combination of LY2510924 with other drugs for gliomas (NCT03746080, NCT01977677, and NCT01288573) and multiple myeloma (NCT00103662, NCT01220375, and NCT00903968) is also under clinical trial.

The microenvironment plays an important role in CSC growth, and it is also a promising target for treatment. Agents targeting the microenvironment in ongoing clinical trials are listed in Table 3.

### CSC-directed immunotherapy

In the early twentieth century, Paul Ehrlich first proposed the idea that an intact immune system suppresses tumor development (advancing cancer therapy with present and Emerging Immuno-Oncology Approaches). Based on the understanding of cellular immune regulation, new methods for cancer treatment have emerged. In addition to the antibodies against the CSC molecules mentioned above, some novel anti-CSC immunotherapeutic approaches, such as immunologic checkpoint blocking or CAR-T cell therapies, have been developed. Some drugs that target the immune checkpoint receptors CTLA-4,^635^ PD-1 (nivolumab,^636^ pembrolizumab,^637^ and cemiplimab,^638^) and PD-L1 (avelumab,^639^ durvalumab,^640^ and atezolizumab^641^) have also been in clinical trials. I ipilimumab, a CTLA-4 antibody, is approved by the FDA, and initial clinical results showed good effectiveness in patients with metastatic melanoma.^642^ For CAR-T cell therapy, as shown in Table 4, CD19, CD20, CD22, CD123, EpCAM, and ALDH have been used for CSC-directed immunotherapy, and most of them are recruited.

**Table 4:** CSC-directed immunotherapy in ongoing clinical trials

Trial description	Condition	Sample size	Phase	NCT Number	Current status
CD19 CAR-T	B cell leukemia and lymphoma	II	80	NCT03398967	Recruiting
CD123 CAR-T	CD122^+^ myeloid malignancies	II	45	NCT02937103	Recruiting
CD22 CAR-T	Recurrent or refractory B cell malignancy	I/II	45	NCT02794961	Unknown
CD22 CAR-T	B-ALL	I	15	NCT02650414	Recruiting
CD33 CAR-T	Myeloid malignancies	I/II	45	NCT02958397	Recruiting
CD33 CAR-T	CD32^+^ acute myeloid leukemia	I	11	NCT03126864	Active, not recruiting
CD38 CAR-T	B-ALL	II	80	NCT03754764	Recruiting
CD138 CAR-T	Multiple myeloma	II	10	NCT03196414	Recruiting
MUC1 CAR-T/PD-1 KO	Advanced esophageal cancer	I/II	20	NCT03706326	Recruiting
EGFR IL-12 CAR-T	Metastatic colorectal cancer	I	20	NCT03542799	Not yet recruiting
MESO CAR-T	Refractory–relapsed ovarian cancer	I/II	20	NCT03916679	Recruiting
MESO-19 CAR-T	Metastatic pancreatic cancer	I	4	NCT02465983	Completed
LeY CAR-T	Myeloid malignancies	I/II	445	NCT02958384	Recruiting
MOv19-BBz CAR -T	Recurrent high-grade serous ovarian cancer	I	18	NCT03585764	Recruiting
LeY CAR-T	Advanced cancer	I	30	NCT03851146	Recruiting
EpCAM CAR-T	Recurrent breast cancer	I	30	NCT02915445	Recruiting
BCMA CAR-T	Multiple myeloma	II	80	NCT03767751	Recruiting

## Conclusions and perspectives

We can conclude that CSCs are a small population of cancer cells that have self-renewal capacity and differentiation potential, thereby conferring tumor relapse, metastasis,^643^ heterogeneity,^644^ multidrug resistance,^645,646^ and radiation resistance.^647^ Several pluripotent transcription factors, including Oct4, Sox2, Nanog, KLF4, and MYC and some intracellular signaling pathways, including Wnt, NF-κB, Notch, Hh, JAK-STAT, PI3K/AKT/mTOR, TGF/Smad, and PPAR, as well as extracellular factors, including vascular niches, hypoxia, TAM, CAF, cancer-associated MSCs, the ECM, and exosomes, are essential regulators of CSCs. Drugs, vaccines, antibodies, and CAR-T cells targeting these pathways have also been developed to target CSCs.^648^ Importantly, many clinical trials on CSCs have also been performed and show a promising future for cancer therapy.

However, there are also multiple hurdles that need to be solved to effectively eliminate CSCs. First, the characteristics of many CSCs in specific types of tumors are not well identified.^649^ Second, since most studies on CSCs are performed in immune-deficient mice in the absence of an adaptive immune system, these models do not recapitulate the biological complexity of tumors in the clinic.^650^ Third, CSCs exist in a specific niche that sustains their survival. However, isolated CSCs are used in most current studies that lacks a microenvironment.^651^ Fourth, the environmental factors in CSC niches are not well understood, and the relationship between TAMs/CAFs and CSCs has not been well studied.^645^ Fifth, since CSCs also share some signaling pathways with normal stem cells, not all the regulatory factors that contribute to CSCs are appropriate for use as therapeutic targets in cancer treatment. Sixth, whether CSCs should be activated or arrested is an open question in cancer therapy.^652^ Seventh, novel signaling and more regulatory levels, such as RNA editing,^653^ epigenetics,^654^ and cellular metabolism,^655^ should be considered in cancer therapy because they also contribute to the stemness of CSCs. Eighth, some inhibitors that target CSC signaling are not very specific, and so new inhibitors need to be designed.^656^ Ninth, natural products that target CSCs should also be studied in the future.^657^ Finally, novel ways of targeting the microenvironment of CSCs are also promising and need to be explored.
